# Painted Reinforced Concrete as an Alternative to Galvanized Steel in Chloride-Rich Environments

**DOI:** 10.1021/acsomega.6c06600

**Published:** 2026-10-07

**Authors:** Helga S. Matera, Elber V. Bendinelli, Felipe G. Nunes, Simone L. D. C. Brasil

**Affiliations:** † Electric Energy Research Center (Cepel), Materials and Mechatronic Department, Rio de Janeiro, Rio de Janeiro 21941-911, Brazil; ‡ School of Chemistry, Federal University of Rio de Janeiro (UFRJ), Rio de Janeiro, Rio de Janeiro 21941-909, Brazil

## Abstract

Galvanization is one of the most common anticorrosive techniques for the protection of metallic structures in atmospheric applications. However, in extremely aggressive atmospheric environments, the material fails to deliver its usual performance, and replacement can be necessary, as in this case study. Painted reinforced concrete has emerged as a promising alternative for extending the service life and enhancing corrosion resistance. They combine the barrier and passivation mechanisms associated with steel reinforcement embedded in concrete with the anticorrosive protection provided by the high-performance coating systems. In this context, this work evaluated the performance of combinations of carbon steel and galvanized steel reinforcement concrete with three commercial coating systems (CS1, CS2, and CS3) under severe conditions expected at the installation site. The test specimens were manufactured using Brazilian CPII cement concrete, and the performance of galvanized steel was assessed in comparison to the expected behavior of carbon steel. Concrete characterization tests were conducted to verify its suitability for the intended application, including water absorption, void index, bulk density, and compressive strength tests, and salt spray exposure tests were performed to assess the anticorrosive performance of the different combinations. Each coating system was individually characterized using techniques to measure its physical and barrier properties, and the reinforcing steels were evaluated for their mechanical properties and chemical compositions. Electrochemical methods, including open-circuit potential monitoring, electrochemical impedance spectroscopy, and polarization resistance, were used to complement the other analyses. The results indicated that the traditional carbon-steel-and-concrete system exhibited a superior anticorrosive performance. Even after long-term exposure to salt spray, the specific influence of each coating system applied to the concrete could not be clearly distinguished, given its excellent anticorrosive performance. Despite this, CS3 consistently showed favorable results in the characterization tests and was therefore selected for the final application.

## Graphical abstract

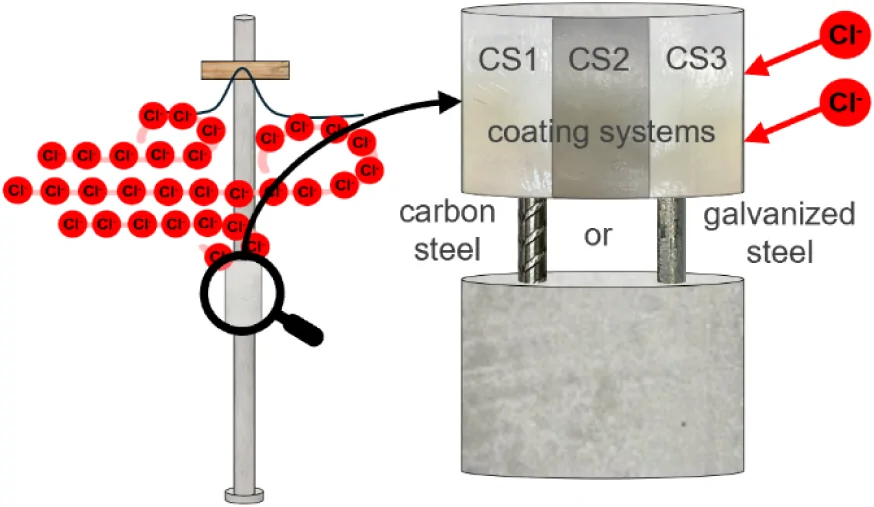

## 1 Introduction

Hot-dip galvanizing is one of the most widely used strategies for protecting steel structures from atmospheric corrosion. By combining cathodic and barrier protection mechanisms, galvanized steel can provide decades of service with minimal maintenance under most exposure conditions.^1,2^ However, its durability may be significantly reduced in extremely aggressive chloride-rich environments, where additional protection strategies become necessary.^3–5^ Although high-performance organic coatings are frequently applied over galvanized steel, there are situations in which even these systems cannot provide the required long-term durability.^2^ Under such conditions, reinforced concrete structures emerge as an attractive alternative for extending the service life.

Reinforced concrete protects embedded steel reinforcement by acting as a physical barrier that limits the ingress of moisture and aggressive species, while simultaneously promoting steel passivation through its highly alkaline pore solution (pH ≈ 12.5).^2,6,7^ Although this combination generally provides higher durability than exposed metallic structures in chloride-rich environments, chloride ions can eventually penetrate the concrete matrix, break the passive film, and initiate rebar corrosion.^8–10^ Therefore, protective coating systems are often employed to further enhance the corrosion resistance and durability of reinforced concrete structures exposed to highly aggressive environments.^2,9^

Organic coatings can be applied directly on the concrete surface or on the reinforcement prior to being cast. Although coating the reinforcement has been extensively investigated, this approach presents several practical challenges, including the need for sufficient abrasion resistance to withstand concrete casting, the possible reduction in bond strength between the reinforcement and the surrounding concrete, and the difficulty of assessing coating integrity after the reinforcement has been embedded.^11–14^ Furthermore, coating the reinforcement hinders the direct interaction between the steel surface and the highly alkaline concrete pore solution and, consequently, the development of the natural passive film that constitutes one of the main corrosion protection advantages of reinforced concrete.

Coating the concrete surface, on the other hand, is not subject to the limitations associated with coated reinforcement, making it an attractive strategy for mitigating pollutant diffusion, water absorption, and chemical attack in reinforced concrete structures. Aguiar et al. evaluated commercial acrylic and epoxy coatings and reported superior performance of the epoxy system, which completely prevented chloride diffusion, significantly reduced mass loss after sulfate exposure, and preserved adhesion after acidic attack.^15^ Similarly, Millán Ramírez et al. found that epoxy coatings provided the highest pull-off strength and the best carbonation protection among the coating systems evaluated.^16^ However, despite their excellent barrier properties, epoxy coatings exhibit poor resistance to ultraviolet (UV) radiation, whereas polyurethane coatings generally offer superior weatherability.^2,17^ Likewise, Pan et al., in their review of concrete surface coatings, highlighted that epoxy coatings provide better abrasion resistance and water resistance, while polyurethane coatings exhibit superior chloride diffusion resistance.^18^ These complementary characteristics suggest that multilayer coating systems may provide enhanced overall performance by combining the advantages of different coating materials.

Despite the extensive research and valuable contributions of previous studies on protective coatings for reinforced concrete, most studies have focused either on coated reinforcement^11–14^ or on a single concrete surface coating,^15,16,18–20^ without assessing the combined potential of multilayer coating systems. In this context, Hussain et al. investigated a multilayer coating system to improve UV resistance and highlighted the benefits arising from the integration of different coating layers.^21^

In addition to carbon steel, galvanized steel has emerged as an attractive alternative for reinforcement in reinforced concrete structures, particularly in marine environments, where its use has increased in Europe and the United States.^22,23^ Like carbon steel, zinc undergoes oxidation in the highly alkaline concrete pore solution, forming calcium hydroxyzincate, a white adherent passive film that protects the reinforcement.^2,24,25^ If there is chloride in the pore solution, the calcium hydroxyzincate is consumed to form zinc hydroxychloride, reducing the passive nature of the film.^24^ Since zinc is less noble than steel, galvanization offers an additional type of protection for the reinforcement, since the galvanized layer needs to be consumed before the onset of steel corrosion.^2,26,27^ Moreover, galvanized reinforcement exhibits higher bond strength to concrete than carbon steel, despite the initial zinc corrosion that occurs upon exposure to the alkaline environment.^11,28^

Electrochemical techniques, particularly open-circuit potential (OCP), polarization resistance (*R*_p_), and electrochemical impedance spectroscopy (EIS), have been widely employed to monitor the corrosion behavior of steel reinforcements in concrete exposed to aggressive environments. These techniques enable the real-time assessment of steel passivation, chloride-induced corrosion, and concrete carbonation.^26,29,30^ However, relatively few studies have combined electrochemical monitoring with accelerated corrosion tests to provide a more comprehensive evaluation of the long-term protective performance of coating systems under aggressive exposure conditions.

This study aims to evaluate the corrosion protection potential of surface-coated reinforced concrete as an alternative to galvanized steel structures exposed to aggressive chloride-rich environments. The research was motivated by a practical case involving a Brazilian transmission line, where the proximity of galvanized steel towers to a potassium chloride (KCl) plant resulted in severe atmospheric corrosion. Based on this case, the study seeks to support the selection of the most suitable construction material and corrosion protection strategy by integrating three well-established approaches—reinforced concrete, galvanized steel reinforcement, and concrete surface coatings—into a single protection system. Galvanized steel reinforcement was evaluated as a substitute for carbon steel, along with three commercial concrete coating systems. Reinforced concrete specimens were exposed to continuous salt spray to simulate a highly aggressive chloride-rich environment. Corrosion processes were monitored using electrochemical techniques, including EIS, *R*_p_, and OCP, while complementary characterization tests were carried out to assess coating performance. The combined results were used to identify the most suitable material and protection strategy for the practical case that motivated this study. To the best of the authors’ knowledge, this is the first study to evaluate the combined corrosion protection provided by galvanized steel reinforcement and commercial multilayer concrete coating systems through integrated electrochemical monitoring and accelerated corrosion testing.

## 2 Methodology

### 2.1 Materials

The concrete specimens were prepared using a sulfate-resistant Portland composite cement CP II-E-RS, which contains 6% to 34% granulated blast furnace slag (GBFS) and is equivalent to ASTM C595 Type IS­(MS).^31,32^ Fine (natural sand) and coarse (crushed granitic stone) aggregates were used, with the sand presenting a medium grading and the crushed stone a fine-grained size. Commercial carbon steel and hot-dip galvanized steel rebar were tested in this work. A stainless-steel bar was used solely as the counter electrode during the electrochemical measurements. Its use was limited to this function, and no assessment of stainless steel as a reinforcing material was intended for this study. The three coating systems used were supplied by three different manufacturers, and each one consisted of a similar three-coat paint system: an epoxy sealer; a modified epoxy–resin intermediate coating; and an acrylic polyurethane topcoat with UV resistance. All paints were supplied with two components (resin and curing agent) and used as received. All chemical reagents used in this work were of an analytical grade.

### 2.2 Preparation of the Concrete Specimens

The mix design chosen for the concrete specimens followed a weight ratio of 1:2:2 of cement, sand, and stone, respectively. The water/cement ratio (w/c) was 0.65 and complied with the mix design I of the Brazilian standard ABNT NBR 6118.^33^ Although this mix design is better suited for low-aggressiveness classes, the use of a C20 concrete mix was intentionally adopted to accelerate chloride ingress and facilitate the evaluation of the corrosion protection performance of the reinforcing steels and concrete coating systems within a practical experimental time frame.^33^ The final mix design consisted of 49.6 kg of cement, 101.5 kg of sand, 99.7 kg of crushed stone, and 32.24 kg of potable water.

The specimens were prepared in accordance with ABNT NBR 5738 and in two different geometries: prismatic specimens without rebars for the adhesion test and with two or three embedded rebars, used respectively for the salt spray and electrochemical tests; and cylindrical specimens for the concrete characterization.^34^ In the three-rebar configuration, the additional reinforcement was a stainless-steel bar acting as the counter electrode for the electrochemical measurements. The prismatic specimens had a 100 mm square cross-section, a 350 mm length, and a 25 mm nominal concrete cover, while the cylindrical specimens had 100 mm in diameter and 200 mm in height.^34^ A total of six cylindrical specimens and 15 prismatic specimens were prepared. Only the prismatic specimens without rebars and those containing two rebars were coated. Figure 1 presents a schematic of the specimens’ geometry and dimensions, as well as the visual aspect of one representative specimen of each shape (Figure 1a,b).

**Figure 1:**
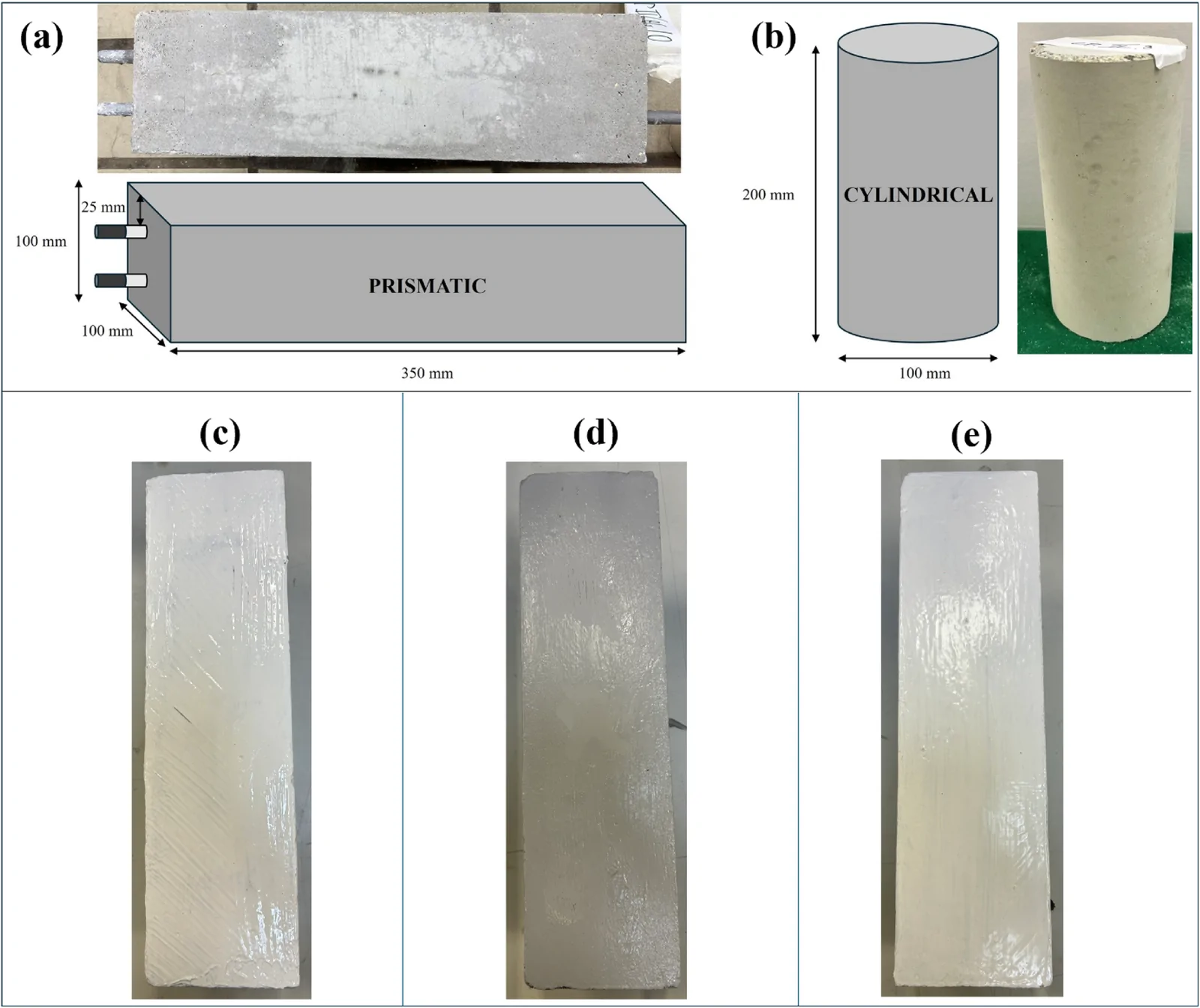
Visual aspect and schematics of the prismatic (a) and cylindrical (b) concrete specimens’ geometry and dimensions; and specimens coated with (c) CS1, (d) CS2, and (e) CS3 prior to any testing.

Although this procedure does not represent the engineering practice for reinforcing steel, the carbon steel reinforcement was subjected to acid cleaning (immersion in a solution of 50% v/v hydrochloric acid with 3.5 g·L^–1^ hexamethylenetetramine) to remove the mill scale and any preexisting corrosion products, thereby standardizing the initial surface condition of the specimens. This procedure facilitated the postexposure visual assessment by ensuring that the observed corrosion resulted exclusively from the accelerated tests. All the rebars were cut to achieve 435 mm of length, guaranteeing that a part of the reinforcement remained concrete-free to allow an electrical contact in the electrochemical measurements. An epoxy-modified coating was applied only at the concrete-air interface to prevent differential aeration, while the remaining exposed end was left uncoated for electrical connection.

The curing stage was conducted for 30 days with partial immersion of the specimens in a saturated calcium hydroxide solution (2.0 g·L^–1^), inside a chamber with a relative humidity of 96 ± 1% and a temperature of 20 ± 1 °C.^34^ At the end of the curing period, the specimens were washed with potable water to remove calcium hydroxide residues and left to dry in controlled conditions.^35^ Finally, 16-grit sandpaper was used to manually sand the specimens’ surface to achieve an adequate surface profile for coating application.

Table 1 contains a brief description and the dry thickness (μm) of each layer (sealer, intermediate, and topcoat) for all three coating systems. The coating systems were named CS1, CS2, and CS3 without mentioning their suppliers since they are commercial products and this work makes a direct comparison between their properties. The coatings were applied with a brush according to their technical data sheets. One prismatic specimen of each reinforcement type, in the two-rebar variation, was left uncoated to act as a control group. Figures 1c (CS1), 1d (CS2), and 1e (CS3) show the visual aspect of one specimen coated with each coating system before any testing.

**Table 1:** Layer Description and Dry Thickness (μm) for All the Commercial Coating Systems (CS1, CS2, and CS3) Used in This Study^a^

coating system	layer	description	dry thickness (μm)
CS1	Sealer	Solvent-free two-component modified epoxy varnish (98 ± 2% VS)	1 coat of 30
	Intermediate	Edge retentive and surface tolerant two-component modified epoxy (85 ± 3% VS)	1 coat of 350
	Topcoat	Styrene-free two-component aliphatic acrylic polyurethane (65 ± 2% VS)	1 coat of 65
CS2	Sealer	Solvent-free two-component epoxy-modified varnish (98 ± 2% VS)	1 coat of 80
	Intermediate	Two-component aluminum epoxy mastic with lamellar fillers and an epoxy-amine resin (74 ± 2% VS)	1 coat of 135
	Topcoat	Two-component acrylic polyurethane cured with an aliphatic isocyanate (63 ± 2% VS)	1 coat of 95
CS3	Sealer	Two-component epoxy polyamine varnish (33 ± 2% VS)	1 coat of 30
	Intermediate	Two-component phenolic novolac epoxy (76 ± 2% VS)	2 coats of 76 each
	Topcoat	Two-component high-thickness aliphatic acrylic polyurethane (68 ± 2% VS)	1 coat of 68

a VS is the abbreviation for volume solids.

The coating systems were also applied to carbon steel substrates, for coating characterization and EIS tests, and as free films, for water absorption and water vapor transmission (WVT) tests. The free films prepared had average thicknesses of 350 ± 40 μm for CS1, 230 ± 20 μm for CS2, and 300 ± 20 μm for CS3. For the abrasion resistance test, two specimens were prepared for each coating system, with thicknesses of 400 ± 10 μm for CS1, 320 ± 10 μm for CS2, and 350 ± 10 μm for CS3. Finally, the average thickness of the substrates prepared for the remaining tests (a total of 6) was 440 ± 60 μm for CS1, 280 ± 40 μm for CS2, and 400 ± 30 μm for CS3.

### 2.3 Steel Characterization and Tensile Testing

Inductively coupled plasma optical emission spectroscopy (ICP-OES) was used to characterize the carbon and galvanized steel rebars chemically. This analysis aimed to classify the steel type according to the American Iron and Steel Institute (AISI) standards and the compositional limits defined by ABNT NBR 7480 for reinforcing steel.^36^ The zinc layer on the galvanized steel rebars was removed prior to the test to avoid analytical interference by immersing them in the same solution used to clean the carbon steel rebars prior to casting. The equipment used was a Thermo Scientific iCAP 7000 series. The test was conducted in duplicate, and the results were expressed as the average chemical composition.

The galvanized and carbon steel reinforcements were also submitted to tensile testing in accordance with ISO 6892-1.^37^ Two specimens with 9.5 mm diameter, 192 mm extensometer gauge length, and a cross-sectional area of 70.88 mm^2^ of each reinforcement type were used. The test was conducted in duplicate and on an Emic DL-3000 Universal Testing Machine and performed under strain-rate control (Method A of the standard), with a crosshead separation rate of 3 mm·min^–1^ during loading and 10 mm·min^–1^ during unloading.^37^ Strain was applied until specimen fracture, and the relevant mechanical properties were calculated in accordance with ISO 6892-1.^37^ The average results were compared against the requirements of ABNT NBR 7480.^36^

### 2.4 Concrete Characterization and Compressive Strength Determination

The prepared concrete was characterized to determine its conformity with the expected properties of a CPII-type concrete.^38,39^ Density, percent absorption, and volume of permeable pore spaces in hardened concrete were determined according to ASTM C642.^40^ Three cylindrical specimens, 4 months after casting, were dried in an oven at 100 ± 5 °C for 72 h. The cooled specimens were weighed to determine their oven-dry mass, designated as *A*, and immediately immersed in tap water at room temperature. After 48 h, the specimens were then placed in a container, covered with tap water, boiled for 5 h, and left to naturally cool overnight. After surface-drying, the specimens were weighed one more time to obtain the saturated mass after boiling, designated as *C*. Finally, the specimens were suspended by a wire in a hydrostatic weighing system to obtain the apparent mass in water, designated as *D*. A 0.01 g precision balance was used in all measurements. The *A*, *C*, and *D* masses were used to calculate the desired properties using the ASTM C642 equations described below, where *ρ* denotes the density of water (1000 kg·m^–3^).^40^ The results were expressed as the average value of each property calculated for the triplicates.

1 $$\mathrm{absorption after boiling}\;\left(\%\right)=\left[\frac{\left(C-A\right)}{A}\right]\times 100$$

2 $$\mathrm{dry bulk density}\;\left(\mathrm{kg}\cdot m^{-3}\right)=g_{1}=\left[\frac{A}{\left(C-D\right)}\right]\times \rho$$

3 $$\mathrm{apparent density}\;\left(\mathrm{kg}\cdot m^{-3}\right)=g_{2}=\left[\frac{A}{\left(A-D\right)}\right]\times \rho$$

4 $$\mathrm{volume of permeable pore spaces}\;\left(\%\right)=\left[\frac{\left(g_{1}-g_{2}\right)}{g_{2}}\right]\times 100$$

The concrete compressive strength test was performed according to the ASTM C39 method.^41^ The test was conducted in the same machine used for the tensile testing, equipped with a 50 kN load cell at a constant displacement rate of 0.2 mm·min^–1^. Three cylindrical specimens, 72 days after casting, were evaluated. The maximum load, in kN, and the specimens’ cross-sectional area, in mm^2^, were used to calculate their compressive strength, in MPa. The characteristic compressive strength (*f*_ck_), in MPa, was calculated according to ABNT NBR 12655 as shown in eq 5, where *σ_rup_* is the arithmetic average of the specimen’s compressive strength and *s* is the standard deviation.^29^ The *f*_ck_ was used to establish the concrete strength class.^42^

5 $$f_{ck}\;\left(MPa\right)=\sigma_{rup}-1.65\times s$$

### 2.5 Characterization of the Coating Systems’ Properties

The coating systems applied to the concrete specimens were characterized to assess their physical and barrier properties. The results obtained were correlated with findings from salt spray exposure and electrochemical measurements to substantiate the selection of the most effective system. Water absorption and WVT tests were conducted to evaluate the coating systems’ barrier properties, while pull-off adhesion and abrasion resistance were used to determine physical properties. Furthermore, EIS measurements were also used to establish the coatings’ barrier effectiveness over time.

The water absorption test was based on the ASTM D570 method.^43^ Three free films of each coating system were left submerged in Type II distilled water during the entire test period. Periodically, the films were surface-dried and weighed on a 0.01 mg precision balance. The experiment was conducted for 434 h, and the results were expressed as mass variation (%), calculated using eq 6.

6 $$\mathrm{Mass variation}\;\left(\%\right)=\left(\frac{\mathrm{final mass}\;-\mathrm{initial mass}}{\mathrm{initial mass}}\right)\times 100$$

The WVT test was based on method B (wet cup method) of the ASTM D1653 standard.^44^ Three free films of each coating system were placed inside permeation cells, ensuring that the only path to water transmission was through the films’ surfaces. The cells were filled with Type II distilled water, put in a desiccator, and periodically weighed on a 0.1 mg precision balance. The masses were used to plot mass change vs elapsed time (permeation curves). Considering a linear region with at least four points of the curve, the WVT rate (g·m^–2^·h^–1^) was calculated using the exposed area of the films (m^2^), the total mass loss in the chosen region (g) and the time in which the loss occurred (h) according to ASTM D1653.^44^ The experiment was conducted for 1946 h.

The pull-off adhesion test evaluated the coating systems’ adhesion and was performed on triplicates of the coatings applied to concrete and carbon steel specimens, based on the methods of ASTM D7234 and ASTM D4541, respectively.^45,46^ For the carbon steel specimens, two conditions were evaluated: initial, in which the specimens were left to cure under controlled conditions; and final, in which the specimens were subjected to 1610 h of salt spray exposure. The pull-off strength was measured using a DeFelsko PosiTest AT-A adhesion tester and a 3 M Scotch-Weld DP460 adhesive. The results were expressed as pull-off strength (MPa) and failure mode (adhesive, cohesive, or substrate failure).

The abrasion resistance test assessed the wear index (*I*) of the coating systems when subjected to friction or dry abrasion. The test was based on ASTM D4060 and executed on a Taber Abraser.^47^ The CS-10 abrasive wheels were used with a 1000 g load per arm. The test was conducted for 2000 abrasion cycles, with weight measurements taken at 500-cycle intervals. The wear index (mg·(1000 cycles)^−1^) was calculated as described in ASTM D4060.^47^

### 2.6 Electrochemical Measurements

To accelerate chloride penetration in the concrete matrix, the prismatic specimens with three rebars used for the electrochemical measurements were subjected to wet–dry cycles adapted from the protocols used by Annaba et al.^48^ and Djenaoucine et al.,^49^ with the following conditions: 5 days of immersion in a 5% w/v NaCl solution; and 2 days of room-temperature drying. The OCP measurements were recorded on the second day of the drying step, while the EIS and *R*_p_ values were made on the fifth immersion day. The measurements were performed for approximately 18 wet–dry cycles.

The OCP monitoring followed the ASTM C876 method for 126 days.^50^ The measurements were made in three equally spaced points (A, B, and C) on each carbon steel or galvanized steel rebar of the test specimen, always on the side corresponding to the 25 mm concrete cover. The working electrodes were the steel rebars, and the reference electrode was the copper–copper sulfate electrode (CSE).^50^ The electrolytic contact between the reference electrode and the concrete surface was achieved with a sponge wet with potable water. The measurements were made with a Fluke 28 II multimeter.

The EIS measurements were performed inside a Faraday Cage, applying a previously defined electrochemical setup, in the two rebars of one specimen of each concrete-reinforcement combination.^51^ A 3.5% w/v NaCl solution was used as the electrolyte. Because of their dimensions, the concrete specimens were only partially immersed in the electrolyte during the measurements, with an active rebar area of 63.4 cm^2^. The carbon or galvanized steel rebar was the working electrode, an Ag/AgCl (sat. KCl) electrode was used as the reference electrode, and the stainless-steel embedded rebar was the counter electrode. Measurements were carried out with an IviumStat.XRe potentiostat, with a frequency range from 100 kHz to 10 mHz, a 10 mV rms sinusoidal amplitude of perturbation around the OCP, and a 5 min OCP stabilization period. In the first measurement, a 1 h stabilization period was observed. EIS was monitored during 12, 19, 41, 56, and 129 days of the wet–dry cycle.

The EIS measurements were also performed on carbon steel-coated specimens to isolate the coating performance without the concrete matrix interference. The same electrolyte, reference electrode, measurement conditions, and potentiostat used for the concrete specimens were also employed in this time. The exposed coated area was 6 cm^2^, and the measurements were made on three spaced points on the same face of the specimens. The carbon steel-coated substrate was the working electrode, and a platinum mesh was used as the counter electrode. The coatings were monitored for 24 h and 5 and 8 weeks of continuous immersion in the 3.5% w/v NaCl solution.

The *R*_p_ measurements in the concrete specimens used the same electrochemical setup and number of replicates employed for the EIS. The scan rate was 0.17 mV·s^–1^ with a polarization of ±20 mV around the OCP. The polarization resistance was determined from the slope of the potential–current curve within ±10 mV of the open-circuit potential. *R*_p_ measurements were made at the same time intervals as the EIS monitoring for concrete samples. Figure 2 shows the concrete specimens immersed in the 5% w/v NaCl solution (Figure 2a), the OCP monitoring on the drying step (Figure 2b), and the setup used for the EIS and *R*_p_ measurements (Figure 2c).

**Figure 2:**
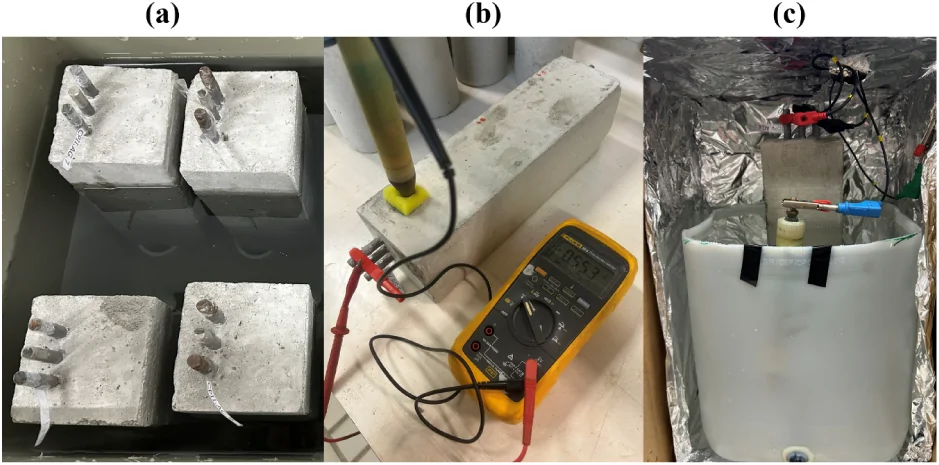
(a) Concrete specimens immersed in the 5% NaCl solution used in the wet–dry cycle; (b) OCP monitoring in the second day of the drying step; and (c) setup used for the EIS and *R*_p_ measurements.

### 2.7 Salt Spray Exposure

The test was based on the methodology described in ASTM B117 and was performed in all the prismatic specimens with two rebars.^52^ A 4–5% w/v NaCl solution was pulverized at 35 °C in the specimens disposed horizontally inside the chamber (Figure 3), with a volumetric flow rate between 1.0 mL·h^–1^ and 2.0 mL·h^–1^. Carbon steel plates coated with the coating systems were also placed in the chamber to evaluate the chloride effect on the systems’ adhesion to the substrate. The concrete specimens were submitted to this test for 2189 h, while the carbon steel specimens were submitted for 1610 h. At the end of the salt spray exposure, the concrete specimens were mechanically fractured to assess the rebars by visual inspection.

**Figure 3:**
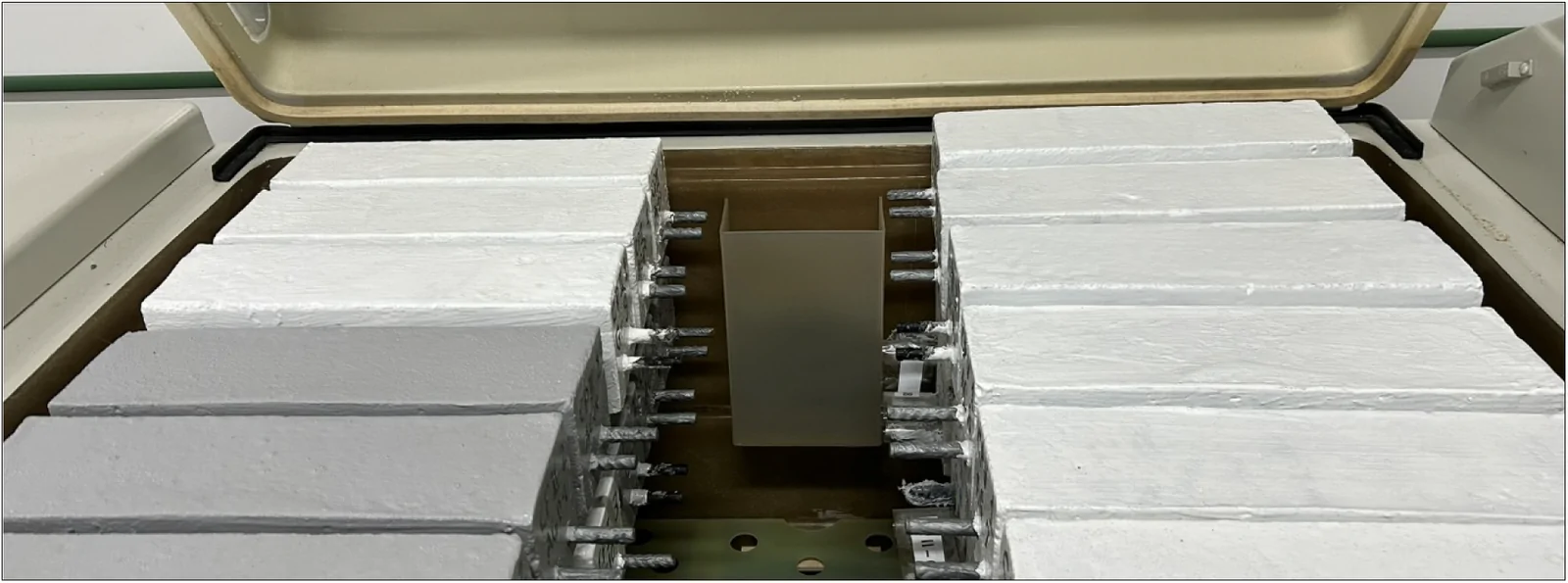
Prismatic concrete specimens in the salt spray chamber before the beginning of the test.

### 2.8 Optical Microscopy

The optical microscopy (OM) test aimed to provide a qualitative analysis of the effects of salt spray exposure and wet–dry cycles on the microstructure of the galvanized steel reinforcement. One rebar embedded in each concrete-coating combination was cut and subjected to metallographic mounting in Bakelite resin, followed by chemical etching with a 2% v/v nitric acid solution to reveal the intermetallic phases of the galvanized coating with higher resolution. For better visualization, the rebars were divided into four quadrants, numbered clockwise. The OLYMPUS GX71 microscope was employed, and the cross-sectional images were obtained at 300× magnification using Stream Motion software.

Table 2 consolidates the tests executed in this study, their overall objective, specimen type used, number of replicates (of each combination), and duration (where applicable).

**Table 2:** All Tests Executed in This Study, with Their Overall Objective, Specimen Type, Number of Replicates (of Each Combination), and Duration (When Applicable)^a^

tests	objectives	specimen type	number of replicates	duration
ICP-OES	Define steel type and composition	AC/AG	2	–
Tensile testing	Evaluate standard requirements	AC/AG	2	–
Density, % absorption, volume of permeable pore spaces	Define the concrete type	CC	3	–
Compressive strength	Define the concrete type	CC	3	–
Water absorption	Barrier properties of the coatings	CSF	3	434 h
WVT	Barrier properties of the coatings	CSF	3	1946 h
EIS	Barrier properties of the coatings	CSAC	3	8 weeks
Pull-off adhesion	Physical properties of the coatings	CSC	3	–
Pull-off adhesion	Physical properties of the coatings	CSAC	6	untested, 1610 h
Abrasion resistance	Physical properties of the coatings	CSAC	2	–
OCP monitoring	Corrosion performance of the reinforcements	CP3	4	126 days
EIS	Corrosion performance of the reinforcements	CP3	2	129 days
*R*_p_	Corrosion performance of the reinforcements	CP3	2	129 days
Salt spray exposure	Corrosion performance of the coating-concrete- reinforcement	CP2	1	2189 h
OM	Qualitative analysis of the galvanized coating	AG, CP2, CP3	1	untested, 2189 h, 129 days

a Abbreviations: AC, carbon steel; AG, galvanized steel; CC, cylindrical concrete specimens; CP*x*, prismatic concrete specimens and *x* is the number of embedded reinforcements; CS*x*, coating system and *x* is the substrate (AC, carbon steel; C, concrete; and F, free films); EIS, electrochemical impedance spectroscopy; ICP-OES, inductively coupled plasma optical emission spectroscopy; OCP, open-circuit potential; OM, optical microscopy; *R*_p_, polarization resistance.

## 3 Results and Discussion

### 3.1 Chemical Composition and Tensile Properties of Steel Reinforcements

Since the transmission line that motivated this study is located in a Brazilian state and, therefore, submitted to Brazilian standards, the discussion of the reinforcing steel’s chemical composition and tensile properties was made using the appropriate Brazilian standards, especially ABNT NBR 7480. The steel’s grading equivalency to international standards is also given, although the discussion limits itself to the ABNT requirements. Table 3 contains the chemical composition, in percentage, of the carbon steel and the underlying steel substrate of the galvanized steel rebars, determined by the ICP-OES analysis. The AISI classifications are AISI 1021 for the carbon steel reinforcement and AISI 1017 for the galvanized steel reinforcement.^53^ Both steel reinforcements comply with the chemical requirements of ABNT NBR 7480, which stipulates maximum contents of 0.35% for carbon, 1.50% for manganese, and 0.05% for phosphorus.^36^

**Table 3:** Average Percentage Chemical Composition of Steel Reinforcements Duplicates Analyzed by ICP-OES

	chemical composition (%)	
element	carbon steel	galvanized steel
aluminum	0.001	0.001
carbon	0.223	0.203
chromium	0.038	0.057
copper	0.077	0.149
manganese	0.607	0.419
nickel	0.037	0.083
phosphorus	0.012	0.005
silicon	0.152	0.079

Figure 4 presents the stress–strain curves obtained from the tensile testing for all the samples, in duplicate for carbon steel and galvanized steel. The carbon steel rebar specimens were denoted as AC1 and AC2, while the galvanized steel specimens were denoted as AG1 and AG2. The galvanized specimens exhibited greater elongation than carbon steel before the fracture point, suggesting a higher ductility for the galvanized steel.^54^ This behavior can be correlated with the steel’s composition since the underlying steel substrate of the galvanized steel and the carbon steel rebars were made from different steel types.

**Figure 4:**
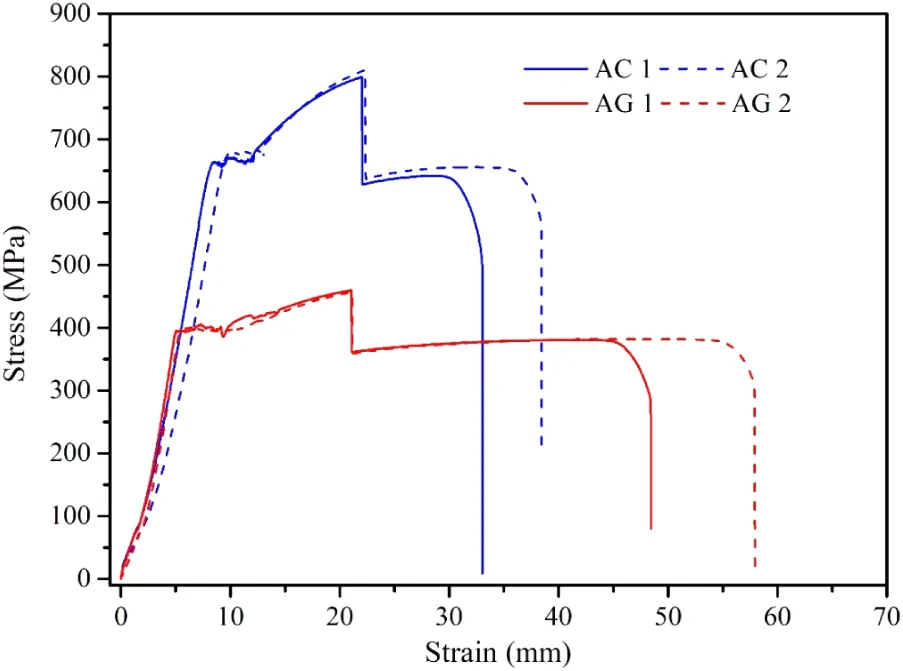
Stress–strain curves for duplicates of carbon steel (AC1 and AC2) and galvanized steel (AG1 and AG2) specimens submitted to tensile testing.

As shown in Figure 4, the elastic-plastic transitions occur abruptly as observed in the yield point phenomenon, which occurs in some types of steel due to alterations in their crystalline structure.^54^ For materials exhibiting the yield phenomena, it is not necessary to employ the offset method, with the yield strength taken as the average stress associated with the lower yield point.^54^ Thus, the tensile strength (*R*_m_), the lower yield strength (*R*_eL_), the Young’s modulus (*E*), and the percentage total extension at maximum force (*A*_gt_) were obtained from the stress–strain curves in Figure 4.^37^ Table 4 contains these properties for both materials.

**Table 4:** Average Tensile Strength (*R*_m_, in MPa), Lower Yield Strength (*R*_eL_, in MPa), Young’s Modulus (*E*), and Percentage Total Extension at Maximum Force (*A*_gt_) Calculated for the Duplicates of Carbon Steel and Galvanized Steel

property	carbon steel	galvanized steel
*R*_m_ (MPa)	826	485
*R*_eL_ (MPa)	663	390
*E* (GPa)	16	18
*A*_gt_ (%)	19	30

The carbon steel rebars exhibited superior tensile strength to galvanized steel, which can be explained by their different chemical compositions. The *A*_gt_ of galvanized steel was higher than that of carbon steel (30% against 19%), confirming the material’s greater ductility. The Young’s modulus was 16 GPa for carbon steel and 18 GPa for galvanized steel, both deviating from the expected value for steel. These differences could be explained by probable slippage at the beginning of the tests commonly occurring with rebars during tensile testing, which does not necessarily imply that the evaluated steels were inadequate.

The *R*_eL_ value classifies the carbon steel as CA-60 grade according to ABNT NBR 7480, equivalent to the ASTM A615 grade 80, which is a steel with high tensile strength (around 600 MPa).^36,55^ The galvanized steel *R*_eL_ value stayed below the CA-50 threshold (500 MPa), equivalent to the ASTM A615 grade 60, the minimum strength category for reinforcement steels.^36,55^ Since the steel compositions are different, as shown in Table 3, it was expected that they could fall into distinct classifications. However, the possible slippage must be considered, which could explain why the galvanized steel failed to meet the tensile requirements for reinforced concrete. Moreover, the galvanized steel rebar purchased lacked ribs, which might have led to greater slippage and a greater impact on their properties.

It is important to emphasize that the tensile results should be interpreted as a function of the chemical compositions of each carbon steel, since it is not expected that the zinc coating would have any meaningful effect on the mechanical properties of the steel rebar.^56–58^ Therefore, if the same carbon steel were used prior to galvanizing, similar results would be expected.^56^

### 3.2 Physical and Mechanical Classification of Concrete

The mean values of absorption after boiling, dry bulk density, and volume of permeable pore spaces for the prepared concrete were, respectively, 6.1 ± 0.2%, 2210 ± 10 kg·m^–3^, and 13.5 ± 0.4%. According to ABNT NBR 8953, the concrete is classified as normal concrete (Type C), with a dry bulk density in the range of 2000–2800 kg·m^–3^.^38^ Helene corroborates this classification by stating that a Type C concrete has a percent absorption in the range of 4.2–6.3% and a porosity (equivalent to the volume of permeable pore spaces) from 10% to 15%.^39^

Figure 5 contains the stress–strain curves for the three concrete specimens subjected to compressive strength testing. The calculated mean compressive strength and *f*_ck_ for the concrete were 23.3 ± 0.9 and 21.8 MPa, respectively. Therefore, the concrete strength class is C20 grade according to ABNT NBR 8953, a Type C concrete with an *f*_ck_ equivalent to 20 MPa that meets the expected values for the chosen mix design.^33,38^ The C20 class is a structural concrete suited for low aggressiveness applications, while higher strength classes, such as C40, are ideal for atmospheres with high chloride contents.^38,42^ As previously stated, a mix design for low aggressiveness was deliberately chosen to allow a better evaluation of the other anticorrosive techniques employed, requiring shorter test times for the electrochemical and salt spray tests.

**Figure 5:**
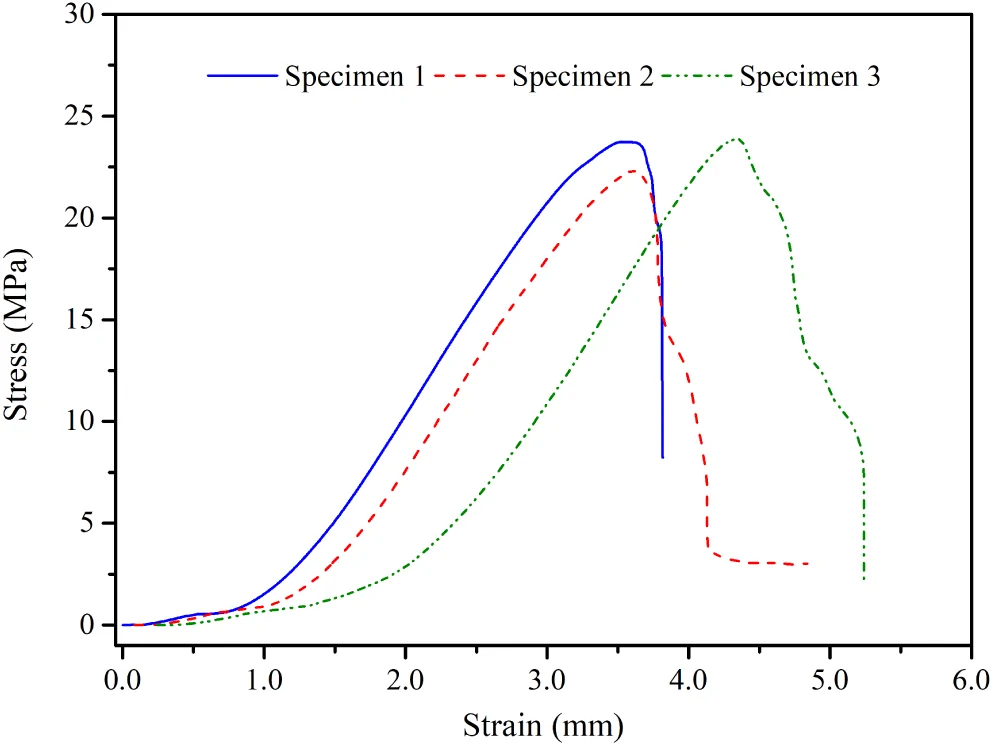
Stress–strain curves for the three concrete specimens submitted to compressive strength testing.

### 3.3 Barrier Properties of Coating Systems by Water Absorption, WVT, and EIS

Since the objective of this study was to compare the performance of commercially available coating systems under their intended application conditions, each coating was evaluated at the dry film thickness recommended by its manufacturer rather than at a common dry film thickness. Figure 6a shows the normalized average mass variation (%) of the coating systems during the water absorption test, with error bars plotted representing the standard deviation of each triplicate. The preservation of a coating’s barrier properties is linked to lower water absorption values since greater water absorption increases the coating capacitance and degrades its dielectric integrity.^59^ CS1 exhibited a slight mass gain at the beginning of the test (considering the standard deviations), followed by a minor weight loss after 200 h. This behavior was probably caused by the dissolution of a soluble compound in the system, most likely in the polyurethane topcoat, or by the leaching of some system’s component. The stabilization suggests that either CS1 did not absorb water after the initial variation or that the rates of absorption and solubilization/leaching remained very alike after 200 h of testing. CS2 and CS3 showed similar performances, with a slight mass gain in the first hours of the test followed by stabilization indicating the free films’ saturation. CS3 films absorbed less water than CS2, indicating CS3 superiority in this test in comparison with CS2. However, based only on the water absorption results, CS1 presented the best features, followed by CS3 and, lastly, CS2.

**Figure 6:**
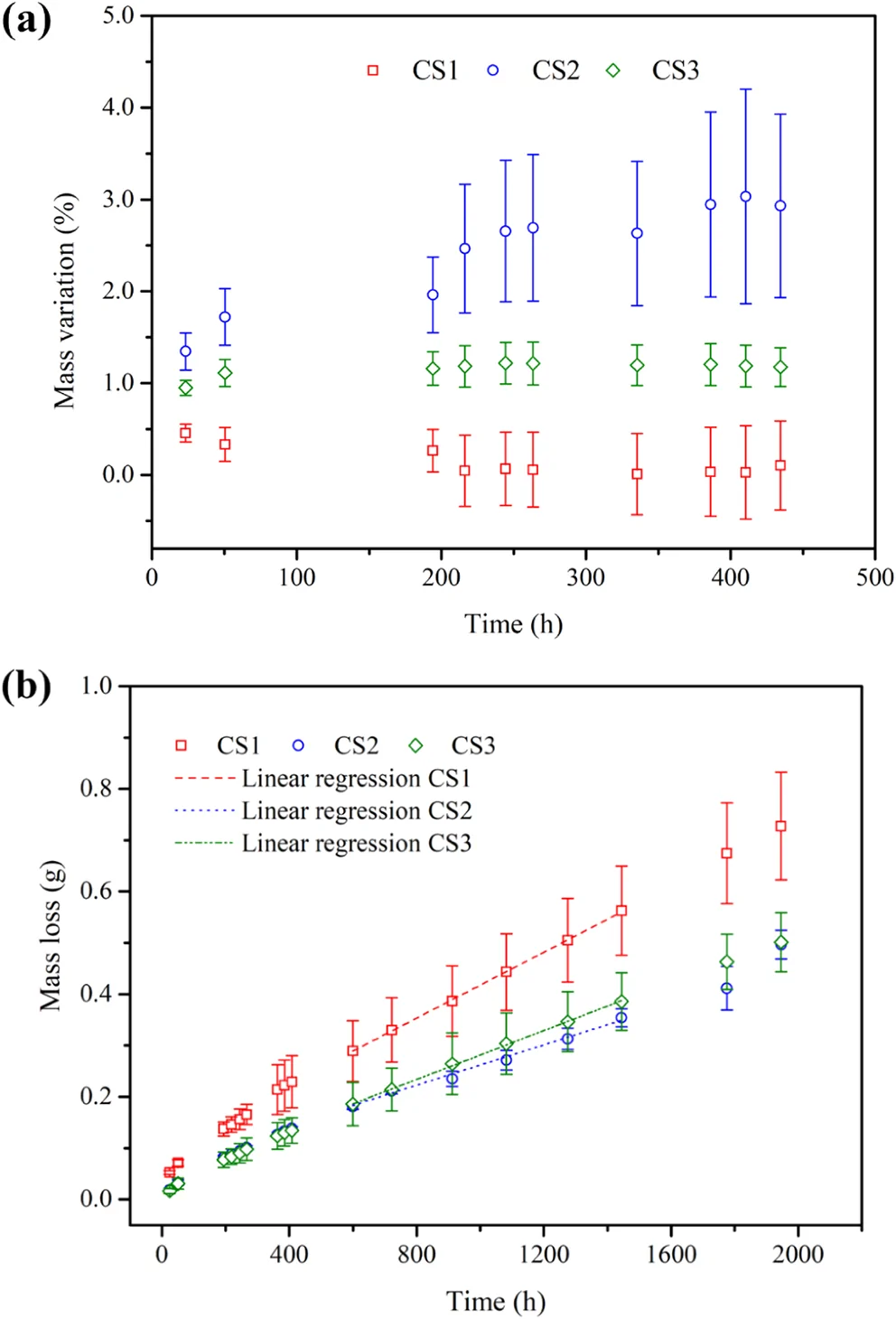
(a) Normalized mass variation (%) for each coating system (CS1, CS2, and CS3) during the 434 h of the water absorption test; and (b) WVT curves for 1946 h of testing for CS1, CS2, and CS3, with the linear regions selected for the WVT rate calculation highlighted.

Figure 6b shows the WVT curves for the coating systems, with the linear regions selected for the WVT rate calculation highlighted. The WVT rates were 0.128 ± 0.003 g·m^–2^·h^–1^ for CS1, 0.078 ± 0.009 g·m^–2^·h^–1^ for CS2, and 0.095 ± 0.003 g·m^–2^·h^–1^ for CS3. As with the water absorption test, higher WVT rates indicate lower barrier properties for a coating.^59^ CS1 presented the worst performance, with a greater WVT rate despite being the thickest system (350 ± 40 μm). The WVT rates of CS2 and CS3 are statistically equivalent when their uncertainties are considered, which makes it impossible to effectively distinguish their performances. Longer testing periods could facilitate the differentiation between the systems, although it is worth noting that CS2 (230 ± 20 μm) showed a similar performance to that of CS3 (300 ± 20 μm) with lower thickness and fewer layers of coat.

Since the impedance responses of the coating systems differed significantly, their barrier performance was comparatively assessed through a qualitative interpretation of the experimental EIS spectra, without equivalent electrical circuit fitting. Figure 7 presents the Nyquist plots for one representative replicate of CS1 (Figure 7a), CS2 (Figure 7b), and CS3 (Figure 7c) as a function of their immersion time in the 3.5% w/v NaCl solution. The Nyquist plots of the other replicates are available in the Supporting Information. A coating impedance analysis can be used to evaluate how aging kinetics and electrolyte immersion time affect its barrier properties.^59^ All of the systems showed a decrease in impedance from 24 h to 5 weeks, which can be explained by electrolyte permeation through the coatings. It usually takes more than 24 h to fill all the conductive paths of the coatings with the electrolyte, leading to the pronounced decline observed.^59^ The measurements made at 5 and 8 weeks stabilized for all three coating systems, suggesting that their barrier properties were not altered with 2 months of immersion. Moreover, the absence of a second capacitive loop in the low-frequency region of the Nyquist plots indicates that no corrosion process occurred during the exposure period.

**Figure 7:**
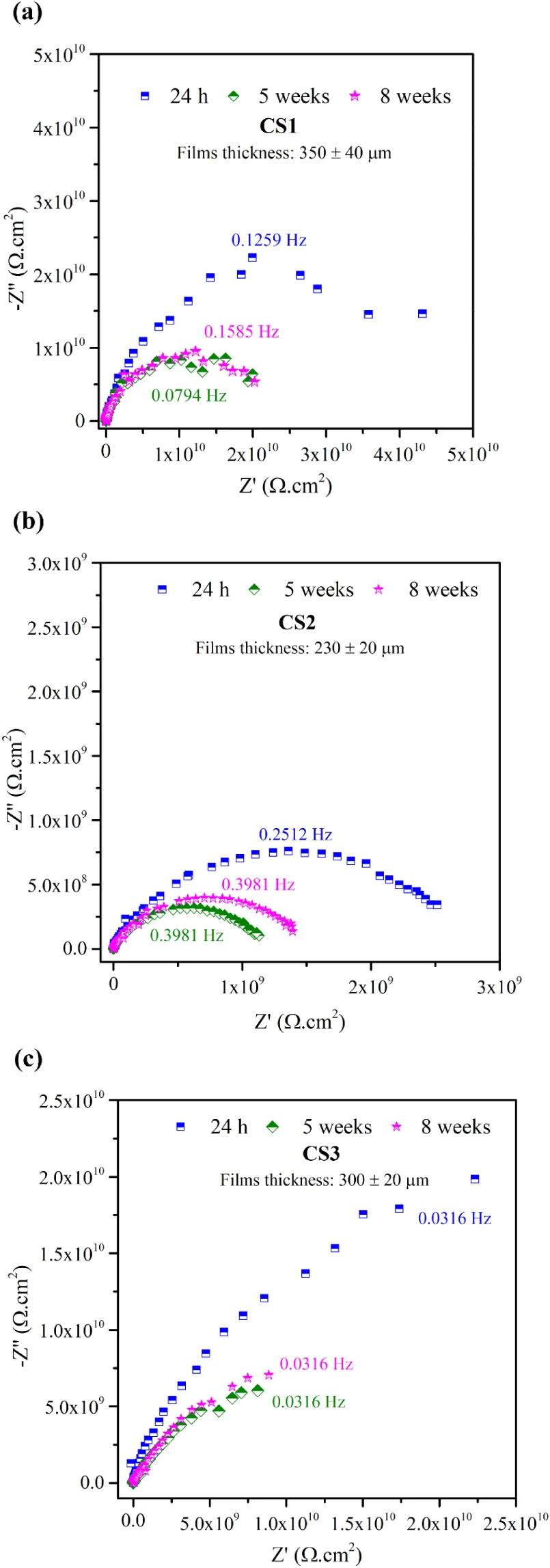
Nyquist plots for one representative specimen of CS1 (a), CS2 (b), and CS3 (c) as a function of their immersion time in the 3.5% w/v NaCl solution. The frequency related to the maximum imaginary impedance is specified in each plot.

CS2 (Figure 7b) impedance values were an order of magnitude lower than those for CS1 and CS3, which may be attributed to their lower thickness. CS3 (Figure 7c) shows a higher initial impedance and a less defined capacitive loop. The capacitive behavior of CS3 indicates less resistive paths for electrolyte permeation, which directly links to the lower water vapor transmission results since CS3 presented the lower WVT.

CS3 exhibited the most significant decrease in impedance magnitude from the first (24 h) to the last (8 weeks) measurement. Since CS3 initial impedance was the highest among the coating systems and no clear capacitive loop was formed, it is logical to assume that a greater decline does not necessarily imply that the system exhibited the poorest barrier properties. Besides, the impedance values remained within the same order of magnitude (10^10^ Ω·cm^2^) and showed little variation from the second to the third measurement, aligning with the good performances of CS3 in the water absorption and WVT tests.

Overall, CS3 exhibited slightly superior barrier properties among all coating systems. In the water absorption test, even with CS3’s intermediate performance (CS1 had the lowest absorption), its total mass gain at the end of the test was still very low (around 0.02 g), suggesting a system’s small tendency to absorb water. The behavior of CS3 in the WVT test was statistically equivalent to CS2 (had the highest water absorption), and once again its WVT rate was very low (0.095 ± 0.003 g·m^–2^·h^–1^). Finally, CS3 showed a higher initial impedance and the absence of a clear capacitive loop in its Nyquist plot, which suggests that even with the greater drop in impedance values during the test, the system was still the most effective in barring electrolyte permeation and protecting the substrate.

### 3.4 Physical Properties of Coating Systems by Pull-Off Adhesion and Abrasion Resistance

Table 5 contains the pull-off strength (MPa) values and failure modes for all of the coating systems applied to concrete and carbon steel (both conditions) substrates. The failure is adhesive when it happens between two layers of the coating system and cohesive when it happens within a layer.^46^ The coating system layers were named based on the standard reference, as follows: A for the substrate; B for the epoxy sealer; C for the modified epoxy resin intermediate; D for the polyurethane topcoat; and Y for the glue.^46^

**Table 5:** Failure Modes and Average Pull-Off Strength (MPa) for the Triplicates of CS1, CS2, and CS3 Applied to Concrete and Carbon Steel for the Initial and Final (after Salt Spray Exposure) Conditions

substrate	condition	coating system	average pull-off strength (MPa)	representative failure mode
concrete	–	CS1	3.7 ± 0.8	100% cohesive A
		CS2	3.7 ± 0.3	
		CS3	3.3 ± 0.3	
carbon steel	initial	CS1	13.2 ± 2	50% adhesive A/B + 30% adhesive C/D + 20% adhesive D/Y
		CS2	12 ± 1	100% cohesive D
		CS3	15 ± 2	50% adhesive D/Y + 50% cohesive D
	final (1610 h of salt spray)	CS1	17 ± 1	60% adhesive A/B + 40% cohesive D
		CS2	11.0 ± 0.3	60% adhesive A/B + 20% cohesive C + 20% cohesive D
		CS3	19 ± 2	30% adhesive D/Y + 70% cohesive

For the concrete specimens, the pull-off strength values could not be differentiated due to the material’s low cohesive strength, which causes the rupture to occur at the weakest point of the system regardless of the coatings applied. Hence, all the concrete specimens presented the same failure mode in the substrate (cohesive A). The pull-off results for the coated carbon steel specimens allowed for a better understanding of the systems’ adhesion.

For the carbon steel in the initial condition, it was once again not possible to differentiate the coatings using the pull-off strength values due to their overlap when the standard deviations are considered. However, the failure modes were clearly distinct, with CS3 presenting the best performance, followed by CS2 and then CS1. CS3 presented equal regions of failure between the glue and the topcoat (adhesive D/Y) and with partial removal of the topcoat (cohesive D), implying great overall adhesion. Although adhesive failures are typically more critical than cohesive ones, the adhesive Y/D occurs when only the glue is removed during the test and does not indicate a system’s poor adhesion, likely occurring as a result of the system having a higher pull-off strength than the glue’s resistance. Adhesive failures between the actual paints (C/D or B/C) or the substrate and the entire coating system (A/B) are more critical, since then a large portion of the coating was removed, directly affecting the systems’ barrier properties and its ability to effectively protect the substrate. CS2 only failed within the topcoat (cohesive D), suggesting that this system also had great initial overall adhesion between its layers and to the carbon steel substrate. CS1’s failure modes in the initial condition were more severe, with regions of complete removal of the coating system (adhesive A/B), and complete removal of the topcoat (adhesive C/D).

After 1610 h of salt spray exposure (final condition), CS1’s failures worsened with a greater area of total removal of the system and more impact on the topcoat. One fixture of CS1 even presented an adhesive A/B failure in the whole test area, suggesting that the salt spray had a great impact on CS1’s adhesion. CS2 also presented a worsening of its adhesion properties, going from a cohesive D failure (partial removal of the topcoat) to a mix of adhesive A/B, cohesive C (total removal of the topcoat and partial removal of the intermediate coat), and cohesive D. Meanwhile, CS3 maintained its failure performance, with only a slight increase in the cohesive D proportion. Since substrate-sealer failure is the most critical one, CS1 showed the worst performance (related to failure mode) among the coating systems. However, it is worth noting that applying a sealer is not recommended for steel substrates, which may have caused the adhesive A/B failures observed.

As for the pull-off strength values in the final condition, the increase observed for CS1 and CS3 can be correlated with the additional curing of the coating systems during salt spray exposure, due to the elevated test temperature (35 °C). CS1 and CS3′s pull-off strength remained statistically equivalent after salt spray, while CS2 presented a slight decrease, as a result of the onset of coating degradation. During application, it was observed that the CS2 sealer was tackier than the others, which could have hindered its curing and compromised its adhesion. Its loss of performance after salt spray exposure also indicates that the system was more susceptible to chloride-rich environments.

Figure 8 shows the evolution of weight loss during the abrasion cycles and the average wear indexes (*I*) for the coating system duplicates. CS2 had the highest *I* and weight loss, while CS1 exhibited the lowest *I* and weight loss, indicating a superior abrasion resistance. The CS3 performance was intermediate compared to the other two systems.

**Figure 8:**
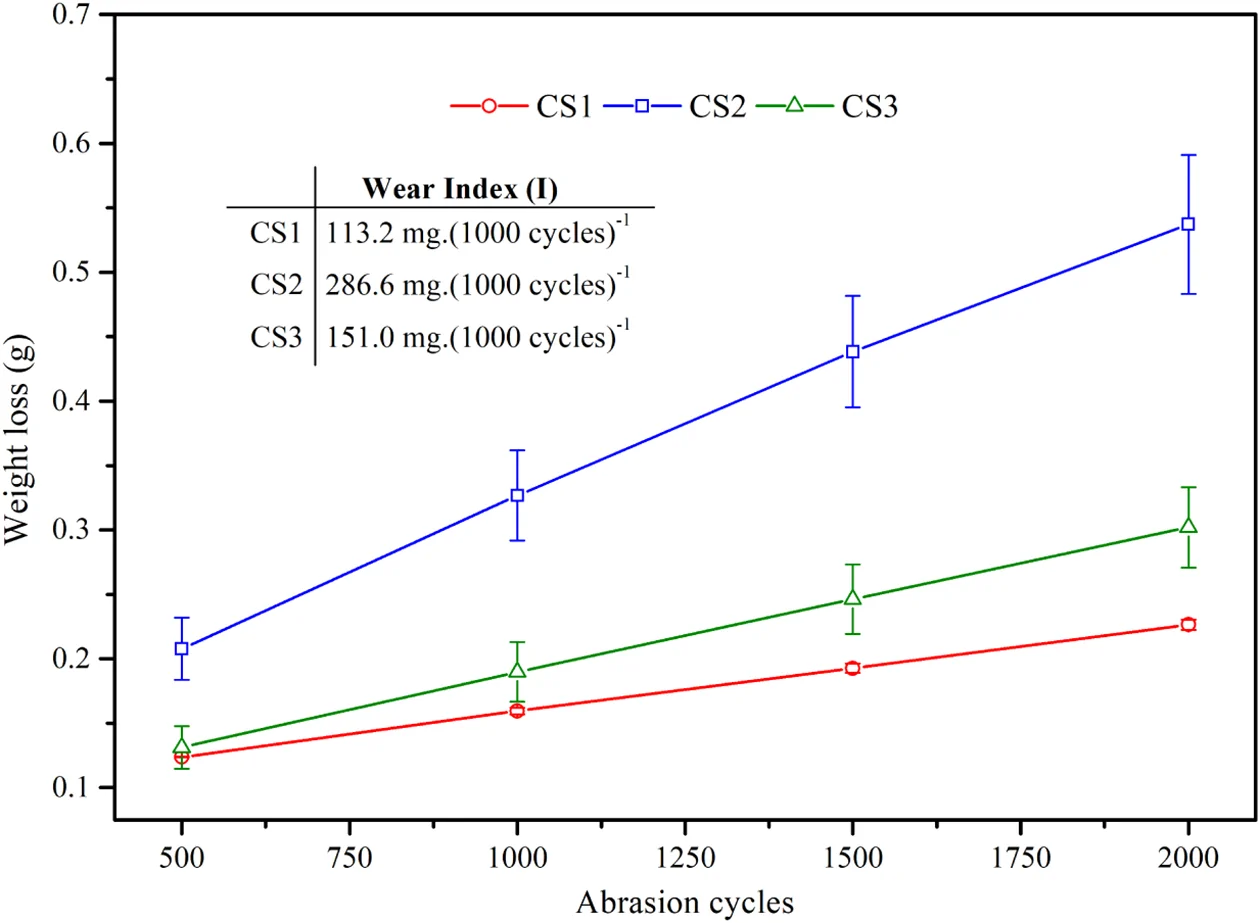
Weight loss during the abrasion cycles and average wear indexes (*I*) for CS1, CS2, and CS3.

The abrasion resistance test is useful for showing how coating systems would resist friction caused by contact with atmospheric particulates and mechanical contacts. In the practical case of this work, the main source of friction is the maintenance activities. CS2, with the highest *I*, would suffer more abrasion and need more maintenance painting, which would increase the cost for the company responsible for the transmission line. CS1 presented the greatest abrasion resistance (lowest *I* and weight loss), with CS3 performance being only slightly inferior.

Table 6 contains a qualitative analysis of the coating systems’ performance in each characterization test, to facilitate their differentiation. The coating systems were comparatively assessed based on the individual interpretation of each characterization test. No weighted scoring model was adopted, since the objective was to discuss the specific contribution of each property to the overall coating performance rather than combine the results into a single performance index. The system with the best performance in a given test is indicated by a plus sign (+), the poorest by a minus sign (−), and the intermediate by a zero (0). Both CS1 and CS3 performed better in two of the five tests analyzed. However, CS3 presented intermediate results in the other tests, while CS1 showed the worst results in two tests, with poor adhesion results and the highest WVT rate. CS2, on the other hand, had the worst overall performance (highest *I*, worst EIS and water absorption results), suggesting that its application would not be recommended.

**Table 6:** Qualitative Analysis of the Coating Systems’ Performance in Each Test, in Which (+) Is a Good Performance, (0) Is an Intermediate One, and (−) Is a Bad One

test	CS1	CS2	CS3
water absorption	+	–	0
WVT	–	+	0
EIS	0	–	+
adhesion	–	0	+
abrasion resistance	+	–	0

### 3.5 Corrosion Performance of Reinforced Concrete by OCP Monitoring, EIS, and *R*_p_

Figure 9 contains the OCP (mV vs Cu/CuSO_4_) monitoring plots during the wet–dry cycles for the carbon (CPII_AC) and galvanized (CPII_AG) steels embedded in concrete. The test was conducted periodically for 18 complete cycles (126 days), and the potential values in Figure 9 correspond to the average of the four rebar measurements. The highlighted corrosion probability regions on Figure 9 were taken from Appendix XI of ASTM C876.^50^ It must be emphasized that the corrosion probability regions only apply to carbon steel.

**Figure 9:**
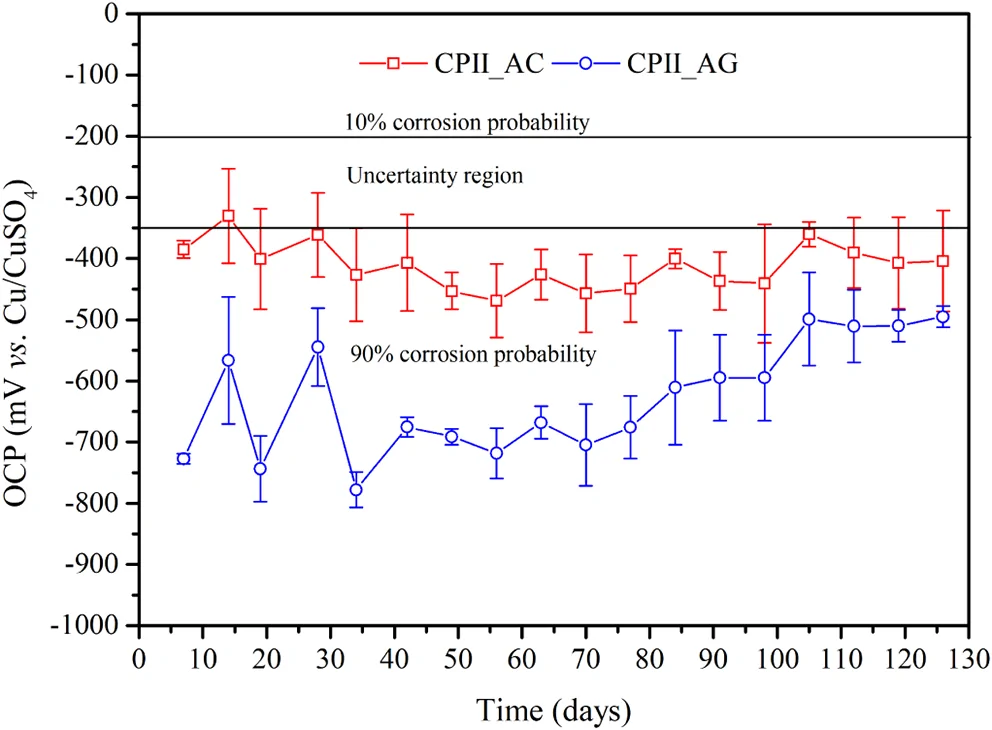
OCP variation (mV vs Cu/CuSO_4_) during the wet–dry cycles in 5% w/v NaCl solution for carbon steel (CPII_AC) and galvanized steel (CPII_AG) embedded in concrete.

The OCP values presented slight fluctuations during the entire test, which were more pronounced in the first cycles, especially for galvanized steel. Santos observed a similar phenomenon in their work when comparing OCP values measured immediately after the specimens’ removal from the immersion solution and after the drying step, with lower potentials registered when the specimens were wet.^60^ Since there were some adaptations in the cycles’ duration in the initial measurements, it is plausible to assume that the shorter drying time caused the lower OCP values observed. Moreover, fluctuations between successive potential measurements can be correlated with the chloride concentration at the rebars’ surface, moisture content, and the formation and subsequent consumption of corrosion products (like zinc hydroxychloride for galvanized steel).^25,61^ Luna Molina et al. also noted that this effect is more pronounced in concretes with Granulated Blast Furnace Slag (GBFS) in their composition, such as the one prepared for this study.^25,31,32^

OCP values for the CPII_AC system exhibited a slight decrease in the initial measurements, which may be attributed to solute (electrolyte) ingress in the concrete matrix and the time required for the electrolyte to actually reach the rebars.^51^ For the remainder of the test, the system behavior remained constant, with the potentials mostly inside the region that indicates a 90% probability of corrosion and occasionally shifting into the uncertainty region. Santos emphasizes, however, that the start of a corrosion process for carbon steel rebars is marked by an accentuated drop in OCP values, a phenomenon that was not detected for the evaluated specimens.^60^ Nevertheless, it is imperative to remember that the regions of corrosion probability from ASTM C876 are only a reference, and the OCP monitoring should be combined with other electrochemical analyses to allow a better understanding of the system’s real status.^39,50,62^

For the CPII_AG system, its potential increases during monitoring, eventually approaching the values observed for CPII_AC in the last measurement. Permeh and Lau noted in their study that the OCP for galvanized steel initially developed to highly negative values, due to the initial corrosion of zinc in alkaline environments, and subsequently increased to the range of −700 to −500 mV, in a behavior associated by the authors with a passivation phenomenon.^27^ Tittarelli and Belleze reported a similar behavior in their work and correlated the formation of the passivation film on galvanized steel with an increase in potential, which shifted toward the range of −600 to −400 mV.^22^ Thereby, it is reasonable to infer that the increase in the potential observed for CPII_AG could have been caused by the passivation of the rebar. Regarding the absolute values of potential, galvanized steel presented an OCP lower than that of carbon steel, as expected, since zinc is less noble (lower corrosion potential) than steel. An increase in the corrosion potential of a zinc-coated system may also be attributed to the corrosion of the zinc layer and the progressive exposure of the Zn–Fe intermetallic phases of the galvanized coating or even the steel substrate.^27,63,64^ However, given the time required for electrolyte permeation and the corrosion resistance of zinc, longer exposure times are expected to expose the Fe-rich surfaces. Therefore, it is more likely that the increase in potential is related to the passivation phenomenon itself or the formation of other corrosion products (not necessarily protective in nature) that change the corrosion potential of the rebar. This hypothesis will be confirmed when the visual aspects of the rebars are evaluated.

Overall, the evolution of the OCP measurements suggests that a corrosion process might have occurred in the CPII_AC system given that the OCP values remained in the corrosion probability region defined by the standard. Nonetheless, the absence of the characteristic decline associated with corrosion in carbon steel and the proximity to the uncertainty region raise the possibility that the rebars could have been passivated as expected. For the CPII_AG system, on the other hand, the increase in potential and the fluctuations between measurements can be linked to the formation of the passivation film associated with galvanized steel embedded in concrete or the formation of corrosion products.

The Nyquist plots for the steel-concrete system provide an understanding of the corrosion behavior for the steel and the concrete matrix, through the division of the plot into two distinct frequency regions.^62,65,66^ The high-frequency region is linked to the concrete response of the system and reflects the resistive character and the porosity of the material, being associated with electrolyte or contaminants (such as chlorides) diffusion into the concrete matrix.^62,65–68^ For any comparison between concrete-steel systems, higher impedance values in the high-frequency region imply that the electrolyte permeation into said system is more difficult, while, analogously, lower impedance suggests that the electrolyte permeation is facilitated.^30,62,66,68,69^ Carbonation and the formation of corrosion products are known to also alter the concrete response; therefore, knowledge of the dominant mechanism is essential to the accurate interpretation of EIS results.^30,62,66,68,69^

The low-frequency region in the Nyquist plot is related to the steel response and can provide information about the development of the passive oxide film on the rebars’ surface.^30,65,68–70^ The steel response part of the Nyquist plot typically exhibits straight-line behavior for passivated systems, with the appearance of a curvature usually indicating depassivation and subsequent corrosion of the rebar.^30,66^ Consequently, the onset of a corrosion process in the steel rebar can be associated with a decrease in impedance values and the formation of a more well-defined capacitive loop in the low-frequency region of the spectra.^30,66,68,70^

Figure 10 shows the Nyquist plots for representative specimens of carbon steel-concrete (10a) and galvanized steel-concrete (10b) systems, as a function of the wet–dry cycles in the 5% w/v NaCl solution. The high-frequency region was magnified to facilitate the visualization. The carbon steel system behavior in the low-frequency region (steel response) remained similar throughout the test period with a strong superposition of the curves. The slight decrease from 12 to 19 days for CPII_AC was probably caused by electrolyte permeation into the concrete matrix, a phenomenon similar to that observed in the EIS of the coated carbon steel specimens.^59^ In the magnified region (high frequencies; concrete response), the impedance values presented a marked decrease and the capacitive loop became more pronounced (smaller diameter) in the 129-day measurement, suggesting that the electrolyte permeation began to be facilitated. It is important to note, moreover, that no indication of a corrosion process was observed in the steel response region of Figure 10a, implying that the facilitated electrolyte permeation might not be related to a depassivation and/or aggravated corrosion of the rebar.

**Figure 10:**
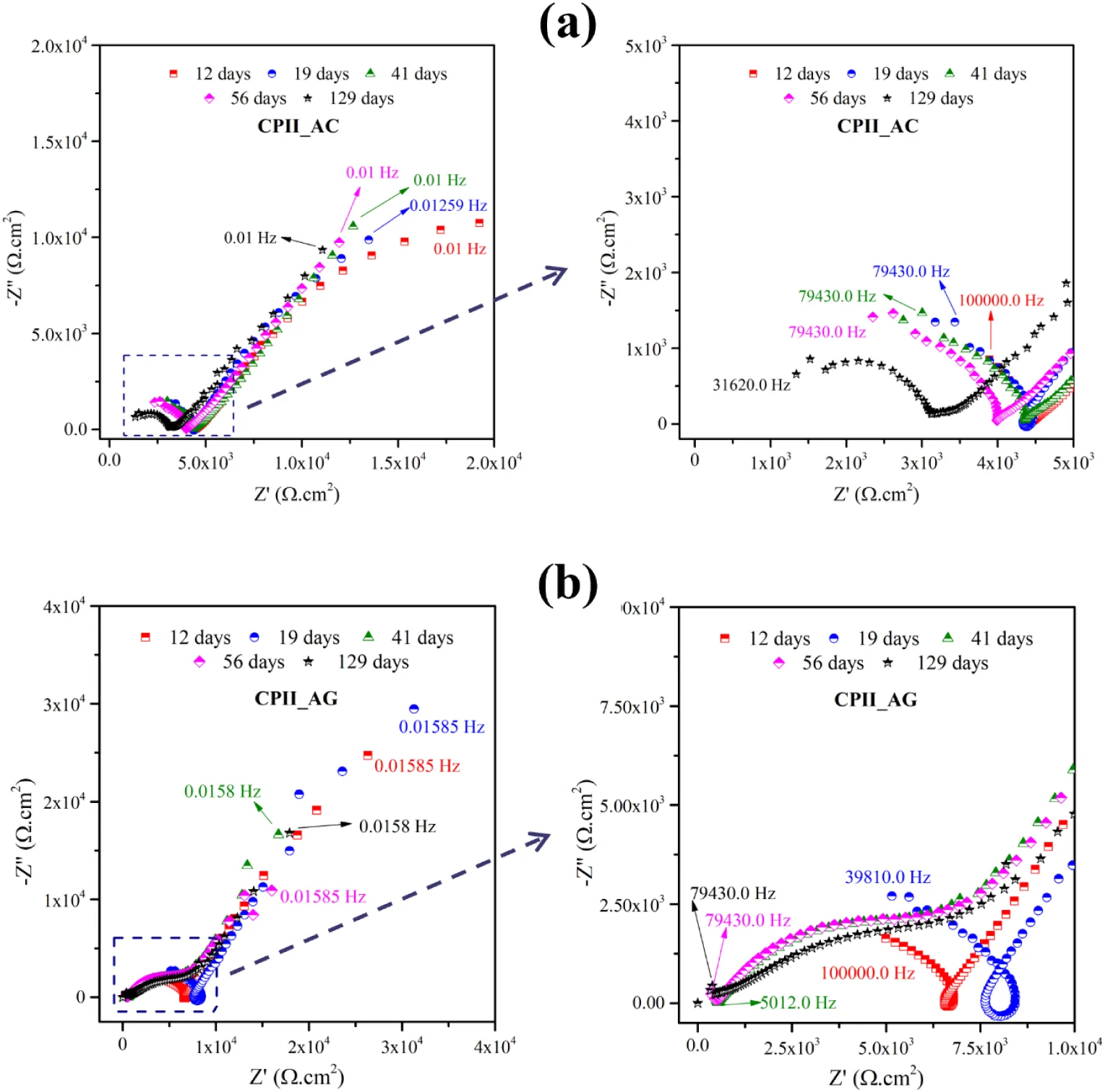
Nyquist plots for representative specimens of carbon steel (a) and galvanized steel (b) embedded in concrete, as a function of their immersion time in the 5% w/v NaCl solution.

For the galvanized steel system, there was also negligible variation in the measurements in the region of the steel response (low frequencies). There is a significant shift in the curve behavior in the magnified high-frequency region for CPII_AG from 19 to 41 days, suggesting that a physical or chemical transition occurred in this system that altered the concrete response. This alteration period coincides with the one observed in the OCP monitoring, which was previously correlated to the formation of the passivation film on the galvanized rebar. A decrease in impedance in the high-frequency region is observed from the 56-day to the 129-day measurement, suggesting, as discussed for carbon steel, that the electrolyte permeation started to be facilitated at the end of the test.

Figure 11 shows the plot of the impedance modulus (|Z|, in Ω·cm^2^) vs immersion time in the 5% w/v NaCl solution for the CPII_AC and CPII_AG systems. The |Z| values were obtained at the fixed frequency of 0.01585 Hz, and the percentage impedance drops were calculated as the normalized difference between the first and last |Z| values for each system. The impedance drops were very alike for the two systems (34.4% for CPII_AG and 32.0% for CPII_AC), suggesting that both reinforcements had a similar interaction with the corrosive medium during the wet–dry cycles. Even with the expected declines, the |Z| values for both reinforcements remained in the same order of magnitude at the end of the test, indicating that the steels did not lose their protection properties after intense chloride exposure. CPII_AG had the highest |Z| values in the low-frequency region (as shown in Figure 11), which is reasonable considering that galvanized steel usually has a better corrosion resistance than carbon steel.

**Figure 11:**
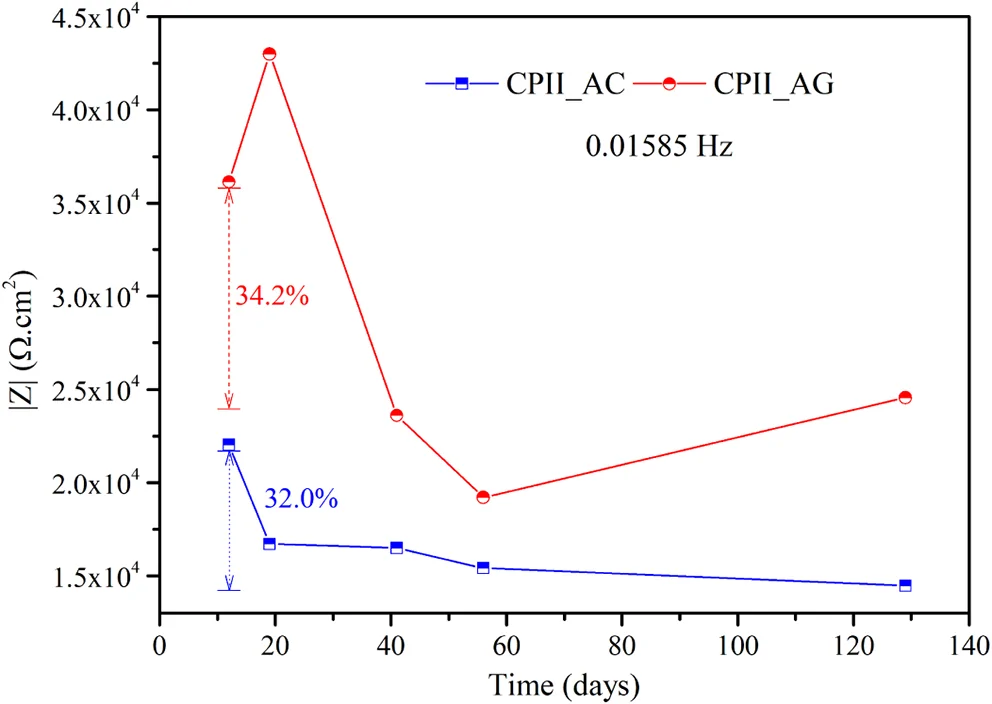
Impedance modulus (|Z|, in Ω·cm^2^) vs immersion time in the 5% w/v NaCl solution for the CPII_AC and CPII_AG systems for the fixed frequency of 0.01585 Hz, with the percentage drops in |Z| values between the first and last measurements highlighted.

Polarization resistance (*R*_p_) is yet another electrochemical technique extremely useful to investigate the corrosion behavior of steel reinforcement embedded in concrete, being commonly interpreted as a measure of the resistance that the concrete-steel system imposes on current flow.^39,62,71^ Polarization resistance results can be discussed either through the *R*_p_ value itself (which is the slope of the potential–current curve) or through a corrosion current density analysis (*i*_corr_), with *R*_p_ and *i*_corr_ being inversely proportional.^39,62,71^ Either analysis provides valuable information about the system’s corrosion rate; however, it is important to remember that *i*_corr_ has a directly proportional correlation with the corrosion rate, and, consequently, *R*_p_ has an inversely proportional one.^39,62^ Since the calculation of *i*_corr_ involves the determination of the Tafel constants whose validity is subject to several restrictions, the sole *R*_p_ analysis was chosen for this study.^39,51^ Assuming similar Tafel constants for both systems, higher *R*_p_ corresponds to lower *i*_corr_.

*R*_p_ changes are frequently accompanied by drastic variations in OCP monitoring. Permeh and Lau evaluated both techniques for galvanized steel and identified that an increase in *R*_p_ (in their study they used *i*_corr_, that, as discussed, decreased) was accompanied by the shift in OCP values associated with the formation of the passive film in the rebar surface.^27^ Hegyi et al. observed a similar trend in their study, in which the authors used *R*_p_ and OCP to assess the effect of galvanization in improving the corrosion resistance of a concrete-carbon steel system.^26^

Figure 12 contains the *R*_p_ values (Ω·cm^2^) for the same specimens evaluated in the EIS test for carbon steel (Figure 12a) and galvanized steel (Figure 12b) embedded in concrete, as a function of the wet–dry cycle in the 5% w/v NaCl solution. The *R*_p_ values for carbon steel remained constant, as shown in Figure 12a, with only slight variations around 30 kΩ·cm^2^. As for the galvanized steel, there was a marked decline in the *R*_p_ values from the first (12 days) to the third (41 days) measurements, followed by a subsequent stabilization. *R*_p_ values for galvanized steel were initially the highest (roughly three times those of carbon steel), suggesting that, in the first weeks, the galvanized-concrete system may have provided superior corrosion resistance. This assumption is corroborated by the higher impedance values observed in the low-frequency region for the system. Furthermore, the stabilization in the measurements also coincides with the moment that the variations in the OCP and EIS monitoring were registered.

**Figure 12:**
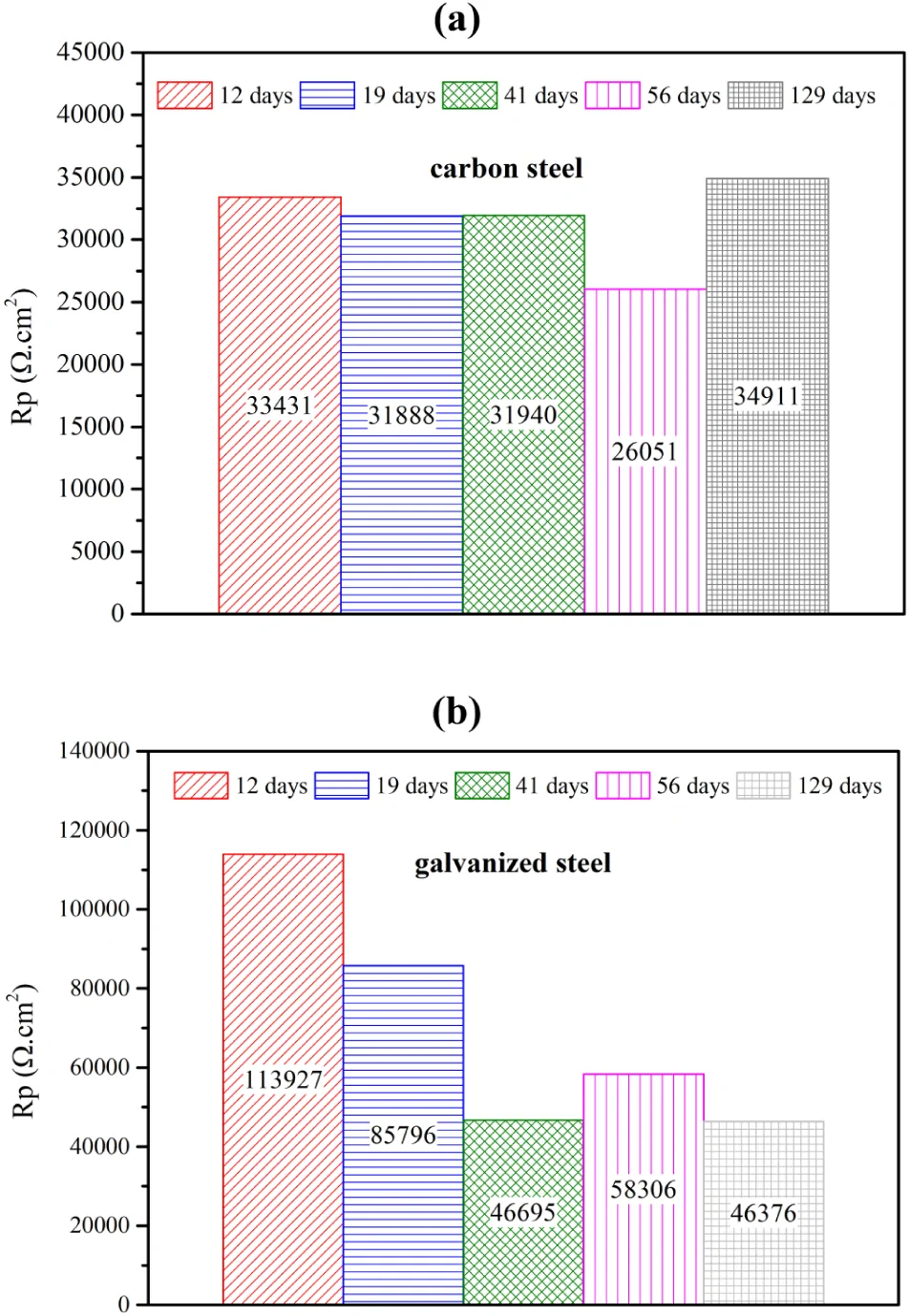
*R*_p_ (Ω·cm^2^) values for one specimen of carbon steel (a) and galvanized steel (b) embedded in concrete evaluated in the wet–dry cycle.

For the concrete-carbon steel system, no significant changes were observed in the *R*_p_ values throughout the test. As for the concrete-galvanized steel system, the consistent decline in *R*_p_ values suggests that a depassivation process might have occurred.^26,27^ At the same time, the OCP values for the system began to rise, in a behavior associated with the formation of the passive film.^26,27,29^ Hence, a thorough analysis of the system after salt spray exposure becomes even more important.

### 3.6 Corrosion Performance by Salt Spray Exposure

Although natural atmospheric exposure provides the most representative assessment of long-term field performance, accelerated corrosion tests are essential for the rapid and standardized comparison of candidate protection systems within a practical experimental time frame. In the present study, the dimensions of the reinforced concrete specimens also limited the use of cyclic weathering equipment, making a continuous salt spray the most appropriate accelerated exposure method for the concrete specimens.

The corrosion assessment of the concrete specimens submitted to salt spray exposure and the wet–dry cycles was primarily conducted by visual inspection of the embedded rebar. To make this possible, the rebar was mechanically separated from the concrete at the end of the tests. None of the evaluated specimens showed significant external alterations during or after these tests, as shown in Figure 13.

**Figure 13:**
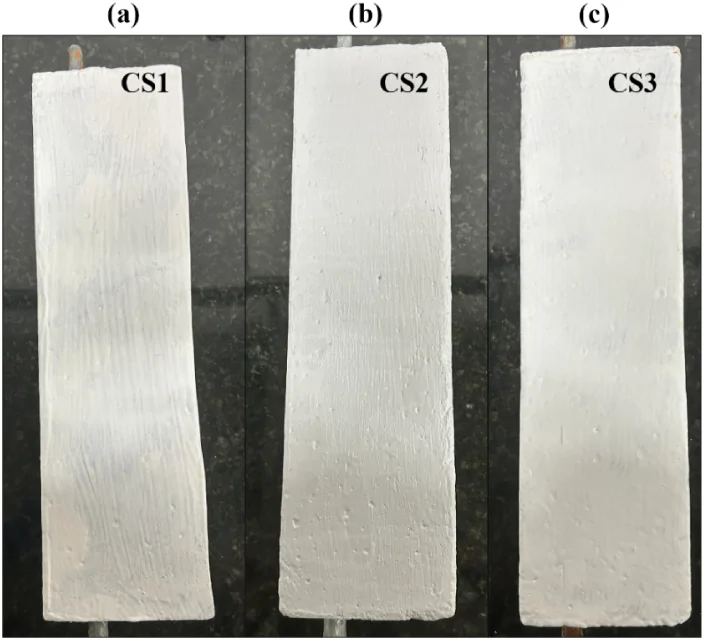
Visual aspect of the prismatic concrete specimens (350 mm length with 100 mm^2^ cross-sectional area) submitted to 2189 h of salt spray exposure (4–5% w/v NaCl, 35 °C, 1.0–2.0 mL·h^–1^) at the end of the test for CS1 (a), CS2 (b), and CS3 (c).

After mechanical fracture, all concrete specimens exhibited a bluish-green coloration, as shown in Figure 14. This phenomenon, known as the greening effect, is commonly observed in concretes prepared with cements containing GBFS, such as the CPII-E-RS cement used in this study.^72,73^ It has been attributed to the hydration and oxidation of sulfide species present in GBFS and has no reported influence on the mechanical properties or corrosion performance of the concrete.^72,73^

**Figure 14:**
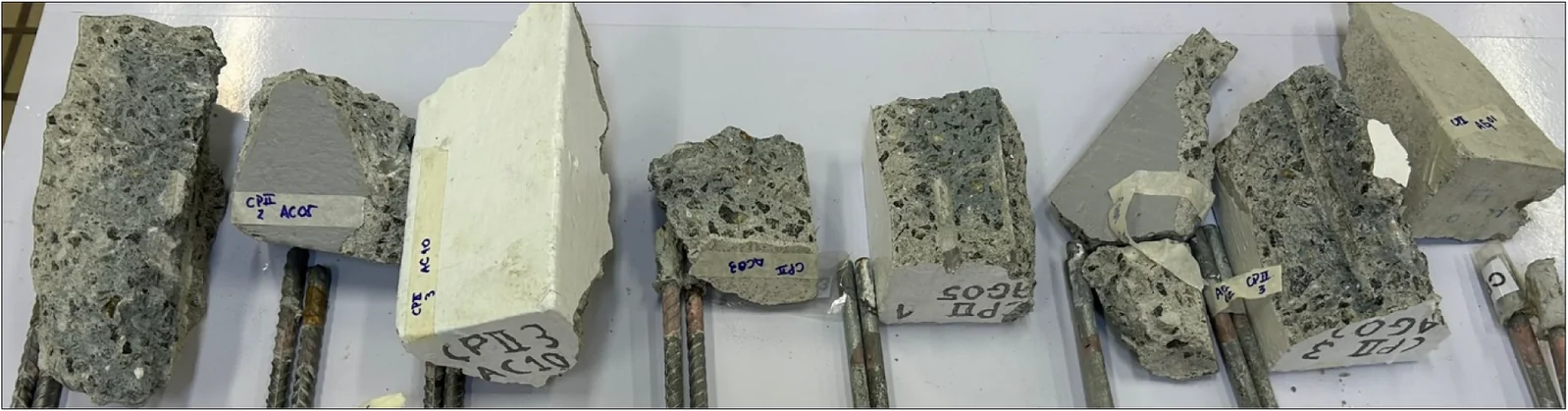
Visual aspect after the mechanical fracture of the prismatic concrete specimens submitted to 2189 h (4–5% w/v NaCl, 35 °C, 1.0–2.0 mL·h^–1^) of salt spray exposure and 129 days of the wet–dry cycles.

Figure 15 contains the photographs taken immediately after the fracture of all the carbon steel rebars embedded in concrete submitted to salt spray exposure and wet–dry cycles. Regardless of the combination of concrete and coating, all of the rebars presented a similar appearance. Corrosion products were only visible in the rebar regions that were not embedded in concrete and, therefore, in direct contact with the salt spray fog or the NaCl solution. The internal parts of the rebars (inside concrete) exhibited no changes from the initial visual aspect accomplished after the precasting cleaning phase. These results support the importance of using OCP monitoring as an auxiliary tool, as the OCP results suggested that the carbon steel rebars were undergoing active corrosion despite their visual appearance suggesting otherwise. Figure 15 also corroborates the well-known anticorrosive potential of the carbon steel-concrete combination, which demonstrated efficacy even in the presence of the highly aggressive chloride-rich environments.

**Figure 15:**
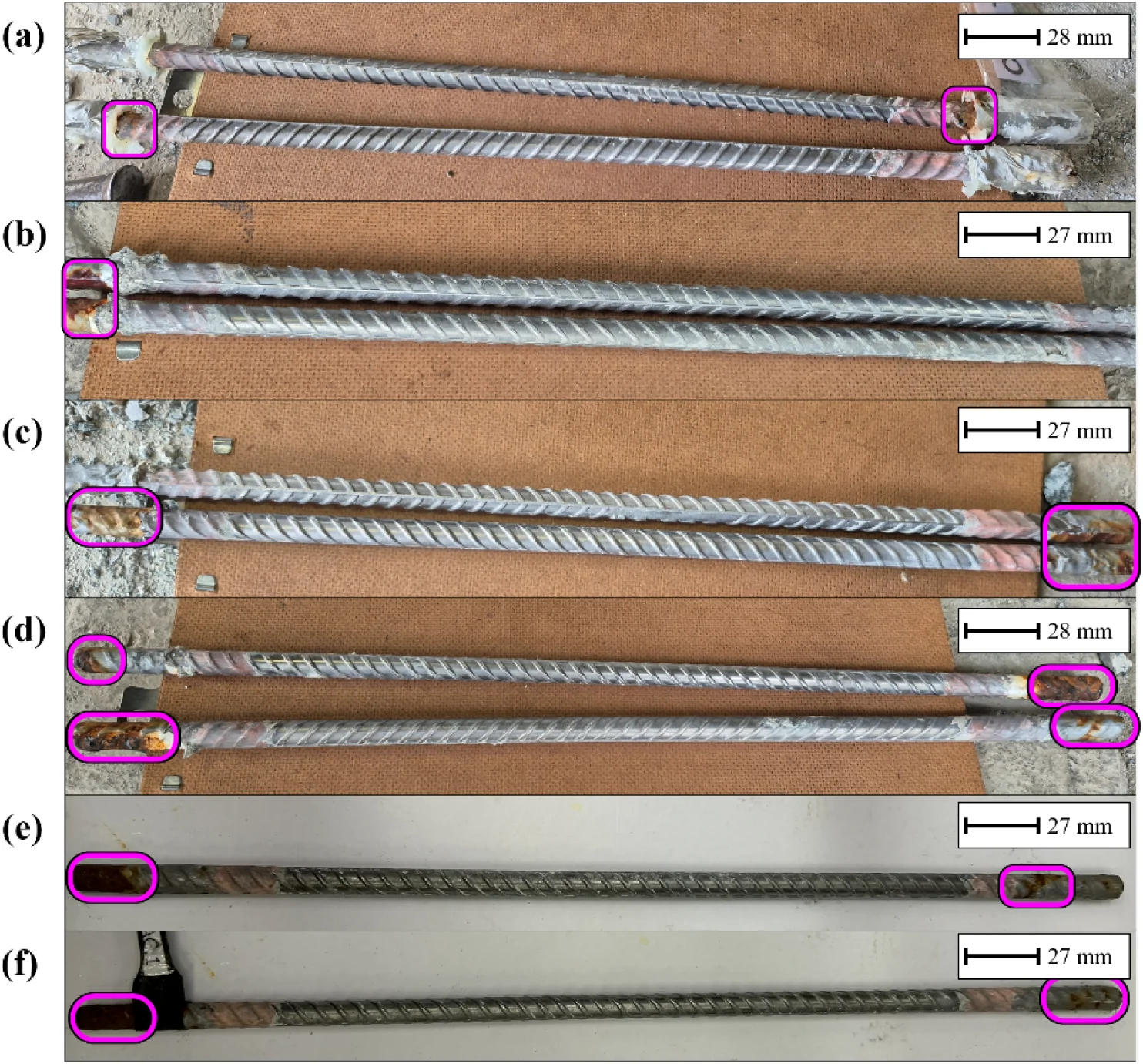
Photographs of the carbon steel rebars (435 mm of length) extracted from concrete specimens after the salt spray exposure (2189 h, 4–5% w/v NaCl, 35 °C, 1.0–2.0 mL·h^–1^) with uncoated (a), CS1 (b), CS2 (c), and CS3 (d) systems; and 129 days of the wet–dry cycles (e and f).

Figure 16 contains the photographs taken immediately after the specimens’ fracture of all galvanized steel rebars embedded in concrete submitted to salt spray and wet–dry cycles. There was widespread corrosion in every rebar, regardless of the combination, with mostly the presence of a white corrosion product. In the external regions of the rebars, this white product was easily removed, which is a characteristic of the oxidation of the *eta* phase on galvanized products. In the internal regions of the rebars, however, both the white and gray products were more adherent to the rebars’ surfaces.

**Figure 16:**
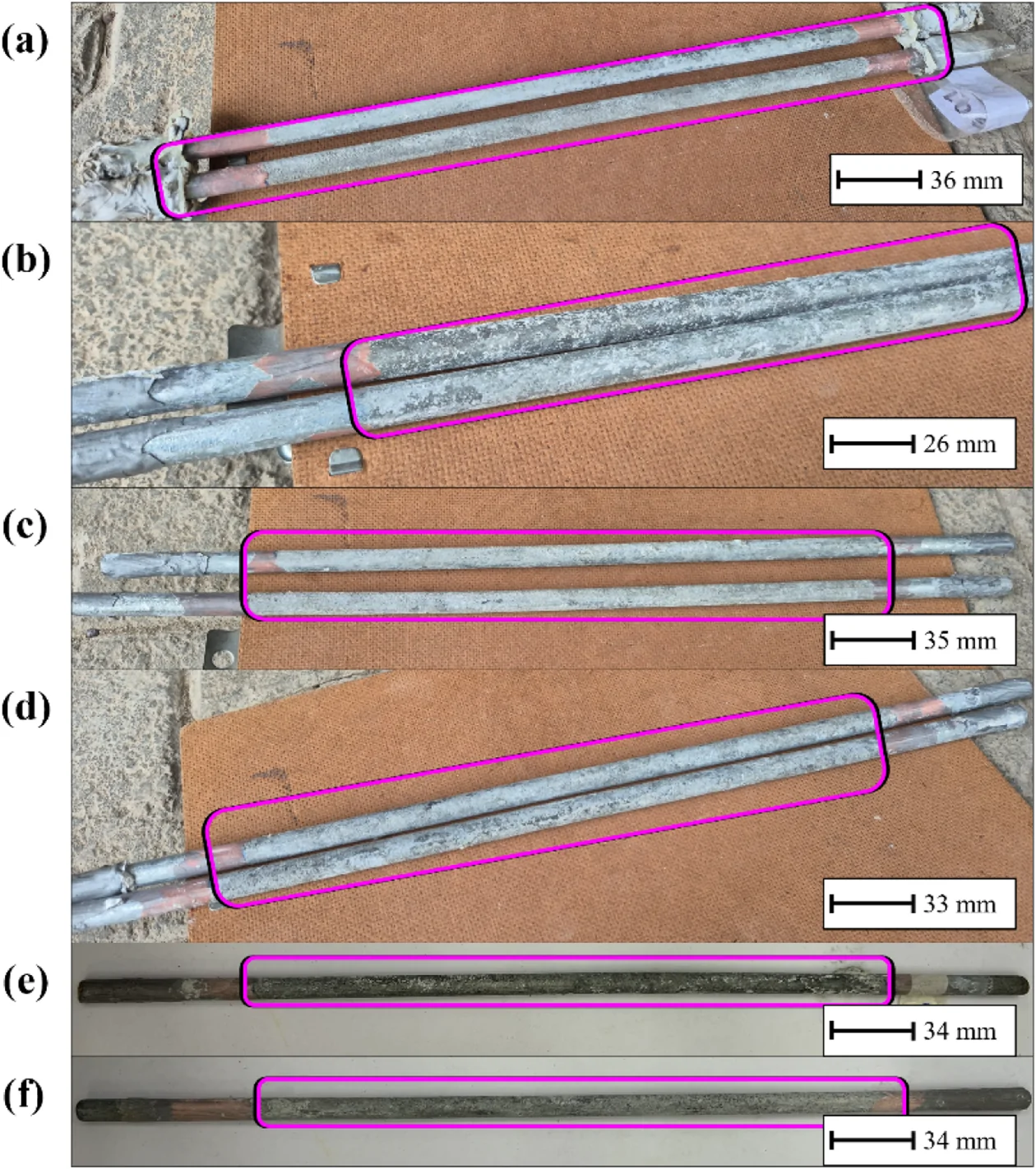
Photographs of the galvanized steel rebars (435 mm of length) extracted from concrete specimens after the salt spray exposure (2189 h, 4–5% w/v NaCl, 35 °C, 1.0–2.0 mL·h^–1^) with uncoated (a), CS1 (b), CS2 (c), and CS3 (d) systems; and 129 days of the wet–dry cycles (e and f).

The presence of white corrosion even in the coated specimens suggests that the amphoteric nature of zinc at the alkaline pH of concrete was responsible for the observed corrosion, rather than the permeation of external aggressive agents, which would have been attenuated by the coatings’ barrier. This hypothesis is supported by Figure 17, in which it is possible to see that the external region of the galvanized rebars (in constant chloride exposure) was better preserved than the internal region, which was in direct contact with the concrete matrix.

**Figure 17:**
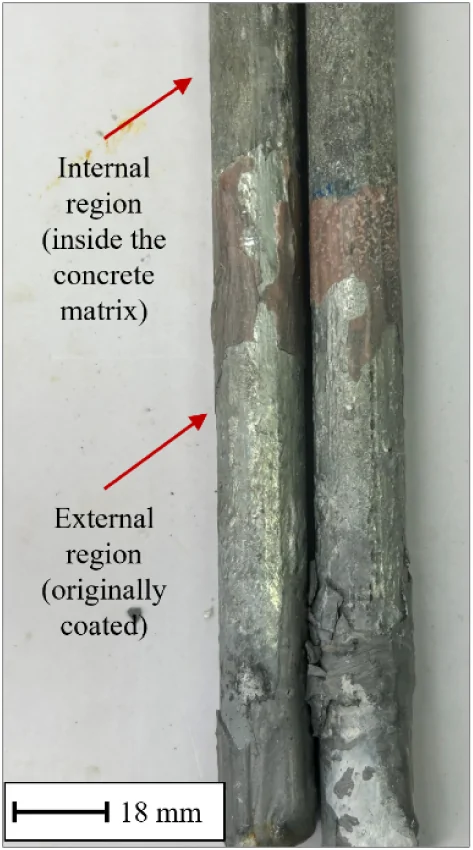
Details of the internal and external regions of a galvanized steel rebar submitted to salt spray exposure (2189 h, 4–5% w/v NaCl, 35 °C, 1.0–2.0 mL·h^–1^), with uniform corrosion in the internal region and with the galvanized layer preserved in the external region.

While the galvanized reinforcement exhibited the formation of white corrosion products, no visible corrosion was observed on the carbon steel reinforcement after accelerated exposure tests. Consequently, a substantially longer exposure period would be required to determine whether the long-term potential protective effect of the corrosion products formed on the galvanized layer provides a measurable advantage over that of conventional carbon steel reinforcement.

### 3.7 Galvanized Coating Analysis by Optical Microscopy

Figure 18 contains the OM images taken at 300× magnification of the two representative quadrants of an untested galvanized steel reinforcement. The galvanized coating is divided into four intermetallic phases, each with a specific proportion of iron: *eta* (η), composed of pure zinc, is the outermost and thickest layer; *zeta* (ζ), with approximately 6% iron; *delta* (δ), with 7–12% iron; and *gamma* (Γ), the closest to the steel substrate, with 21–28% of iron.^2^ In Figure 18, the galvanized phases were well-defined. It is interesting to note that the galvanized coating thickness varied significantly throughout the reinforcement (in the measured range from 84.94 to 140.16 μm), which implies that the galvanization process was not uniform.

**Figure 18:**
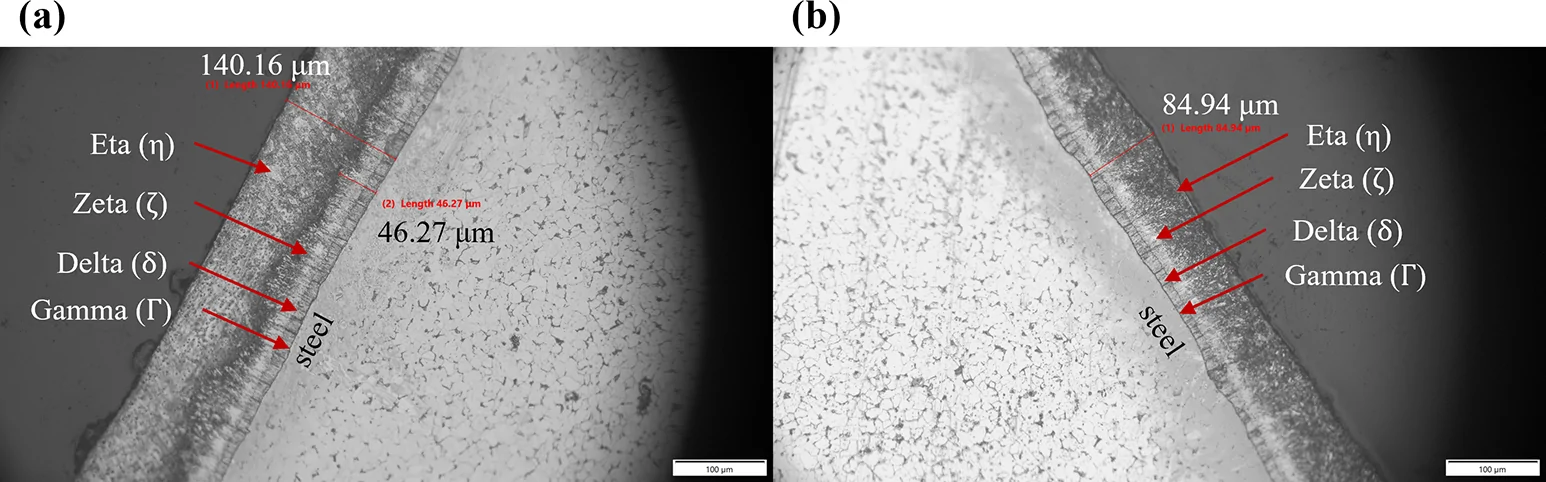
OM images taken at 300× magnification of the first (a) and second (b) quadrants of the untested galvanized steel reinforcement. The galvanized phases *eta* (η), *zeta* (ζ), *delta* (δ), *gamma* (Γ), and the coating thickness (μm) are highlighted.

Figure 19 shows the OM images of one quadrant of the galvanized steel reinforcements after 2189 h of salt spray exposure for the uncoated (Figure 19a) and coated with CS1 (Figure 19b) specimens. Figure 19b is representative of the specimens coated with CS2 and CS3, as they showed no observable differences in the OM images. The galvanized coatings of the rebars embedded in the uncoated (Figure 19a), coated with CS1 (Figure 19b), and with CS3 (Figure 19b) concrete specimens show, once again, no clear differentiation between the coating systems. All phases of the galvanized coating remained well preserved after salt spray exposure. Although white corrosion products indicated superficial corrosion of the η phase, no apparent coating consumption was observed by cross-sectional optical microscopy. Similar white corrosion products were already present on the galvanized rebars before salt spray exposure as a result of the interaction with the concrete pore solution, and no noticeable increase was observed after the test, suggesting that corrosion remained superficial. Furthermore, considering the natural variation in zinc coating thickness, no measurable reduction in coating thickness could be attributed to corrosion.

**Figure 19:**
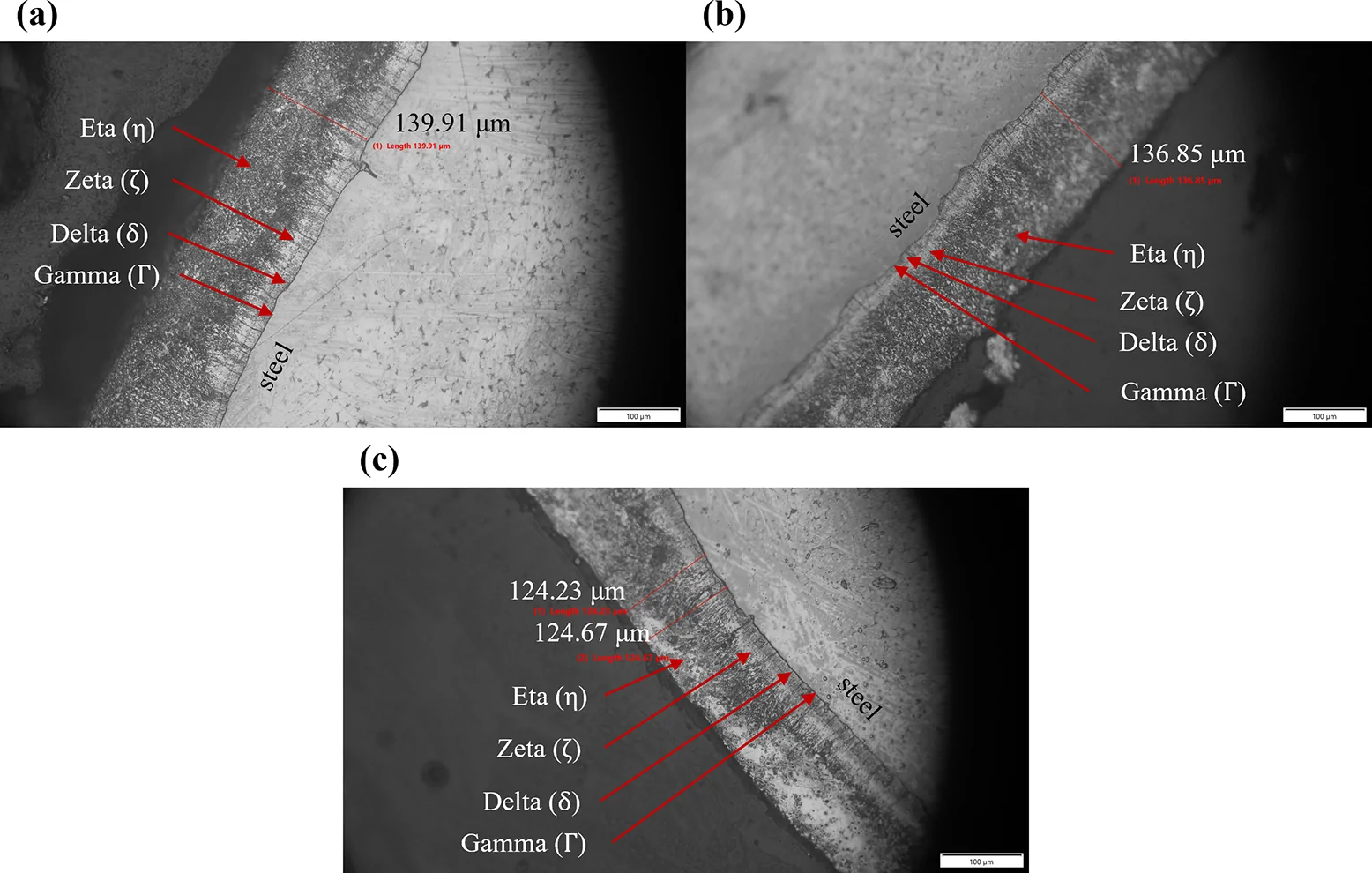
OM images taken at 300× magnification of one quadrant of the galvanized steel reinforcements embedded in the uncoated (a) and coated with CS1 (b) concrete specimens after 2189 h of salt spray exposure (4–5% w/v NaCl, 35 °C, 1.0–2.0 mL·h^–1^); and of the representative specimen of the electrochemical measurements after 129 days of the wet–dry cycle (c). The galvanized phases eta (η), zeta (ζ), delta (δ), gamma (Γ), and the coating thickness (μm) are highlighted.

Figure 19c shows the OM image taken at 300× magnification of one quadrant of the galvanized reinforcement evaluated in the EIS and *R*_p_ tests (after 129 days of the wet–dry cycle). As in the uncoated and coated specimens of the salt spray, there are no apparent changes in the phases of the galvanized coatings.

Since EIS and *R*_p_ did not suggest the formation of the passive film, it is not possible to affirm that the corrosion product was indeed this film. Moreover, the presence of a gray adherent deposit, more associated with the attack of the Fe–Zn phases of the galvanized coating, also raises doubts about the true passive nature of the corrosion products formed. Extending the salt spray exposure and the electrochemical monitoring would allow a better understanding of the underlying corrosion mechanism.^24^ Furthermore, Luna Molina et al. identified an increased risk of depassivation of galvanized steel rebars embedded in concrete containing GBFS, such as the one prepared, which may have hindered the formation of the passive film.^25^

It is also worth noting that the galvanized steel corroded faster than carbon steel, which can be explained by the system’s reactivity in alkaline media. As shown by zinc’s Pourbaix diagram, the *eta* phase has a natural tendency to corrode in both alkaline and acid media.^2^ In long-term applications, galvanized steel could still exhibit a longer service life, since the entire layer needs to be consumed before the underlying carbon steel begins to be corroded. However, since this study combined the reinforced concrete with anticorrosive painting, and the traditional carbon steel showed a great performance, the chosen rebar material for the cited application was, indeed, carbon steel.

As discussed, the coated CS1, CS2, and CS3 systems and the uncoated reinforced concrete systems exhibited no significant differences at the end of salt spray exposure. It is, nevertheless, expected that the coated specimens would start to show a superior corrosion resistance with longer test periods.^8–10,15^ Since CS3 showed the best performance in the characterization tests, this system was selected for practical application.

## 4 Conclusions

Based on the conditions evaluated in this study, carbon steel reinforcement proved to be a superior option to galvanized steel for the manufacture of concrete reinforced poles. The mechanical and chemical characterization confirmed that the carbon steel used was within specifications, while galvanized steel’s tensile strength did not meet the expected values for use as reinforcement (due to the chemical composition of the underlying carbon steel). The prepared concrete properties were within all specified ranges, and it was classified as a normal concrete with a strength class C20. Higher strength classes, such as C40, are preferred for applications in extremely aggressive atmospheres. As for the coating systems, it was not possible to differentiate their performance based on salt spray exposure. Their characterization tests showed greater adhesion and barrier properties for CS3. The OCP monitoring, compared with the existing literature, suggested the formation of a passive film on galvanized steel rebars, as evidenced by the removal of the reinforcement from concrete which showed a widespread white corrosion. The EIS and *R*_p_ tests, however, indicated that the product did not exhibit passivation characteristics. Overall, the results of this study showed that the carbon steel-concrete combination performed better under practical conditions and was, therefore, the chosen as the solution.

Although none of the coating systems provided a significant improvement in the specimens’ corrosion resistance, the coating of the poles is still recommended due to the application of a chloride-rich environment and a history of failed solutions. Among the systems tested in this work, CS3 exhibited a superior overall performance and was recommended for pole manufacturing to extend their service life. It is also recommended that the coating be applied before pole installation to minimize chloride deposition on its surface, which can cause coating defects.

While the results provide consistent technical support for the selection of the carbon steel reinforcement combined with the CS3 coating system under the conditions investigated, a definitive recommendation for field application should also consider long-term service life prediction and additional environmental exposure conditions beyond those evaluated in the present study.

## Supplementary Material



## Notes

Nyquist plots of the other replicates used in the EIS measurements for the coating systems and concrete-reinforcement systems (PDF)

## §

Polytechnic School, University of São Paulo (USP), 05508-010, São Paulo, São Paulo, Brazil
