# Supplementary material for: Painted Reinforced Concrete as an Alternative to Galvanized Steel in Chloride-Rich Environments
Source: ACS Omega. 2026 Oct 7;11(41):63245–64. doi: 10.1021/acsomega.6c06600 (PMC13688579; doi:10.1021/acsomega.6c06600)
Supplement: Supplementary file 1 [file ao6c06600_si_001.pdf]

Supporting Information

Painted Reinforced Concrete as an Alternative to Galvanized Steel in Chloride-rich Environments

AUTHOR NAMES : Helga S. Matera[a,b], Elber V. Bendinelli[a], Felipe G. Nunes[a,†], Simone L. D. C. Brasil[b].

[a]Electric Energy Research Center (Cepel), Materials and Mechatronic Department, 21941-911, Rio de Janeiro, Rio de Janeiro.

[b]School of Chemistry, Federal University of Rio de Janeiro (UFRJ), 21941-909, Rio de Janeiro, Rio de Janeiro, Brazil.

Figure S1 contains the Nyquist plots for the other two replicates of CS1 (S1a), CS2 (S1b), and CS3 (D1c) as a function of their immersion time in the 3.5% w/v NaCl solution. Figure S2 contains the Nyquist plots for the other replicate of the AC-concrete (S2a) and AG-concrete (S2b) systems as a function of their immersion time in the 3.5% w/v NaCl solution. The frequency related to the maximum imaginary impedance is specified in each plot.

Figure S1. Nyquist plots for the other two replicates of CS1 (a), CS2 (b), and CS3 (c) as a function of their immersion time in the 3.5% w/v NaCl solution. The frequency related to the maximum imaginary impedance is specified in each plot.

(a)

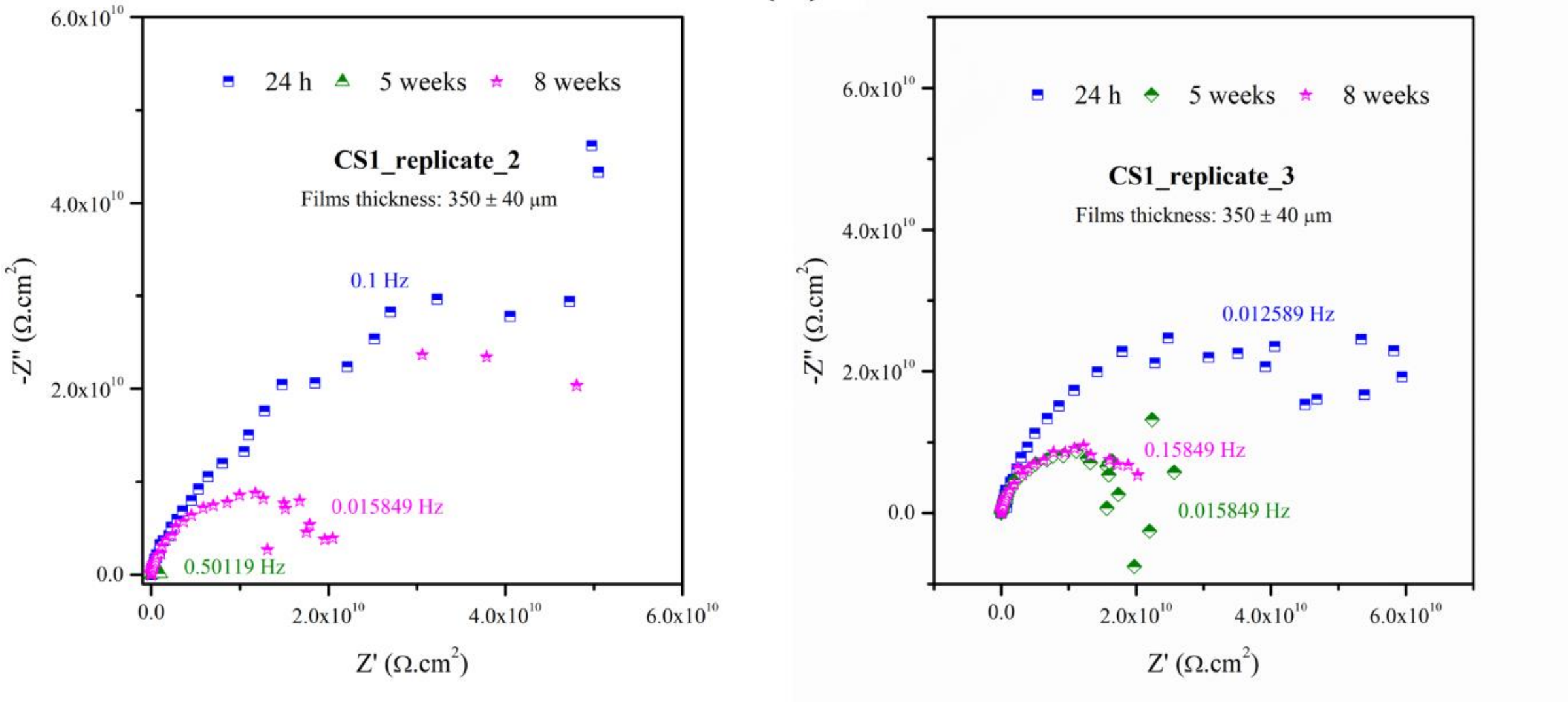

(b)

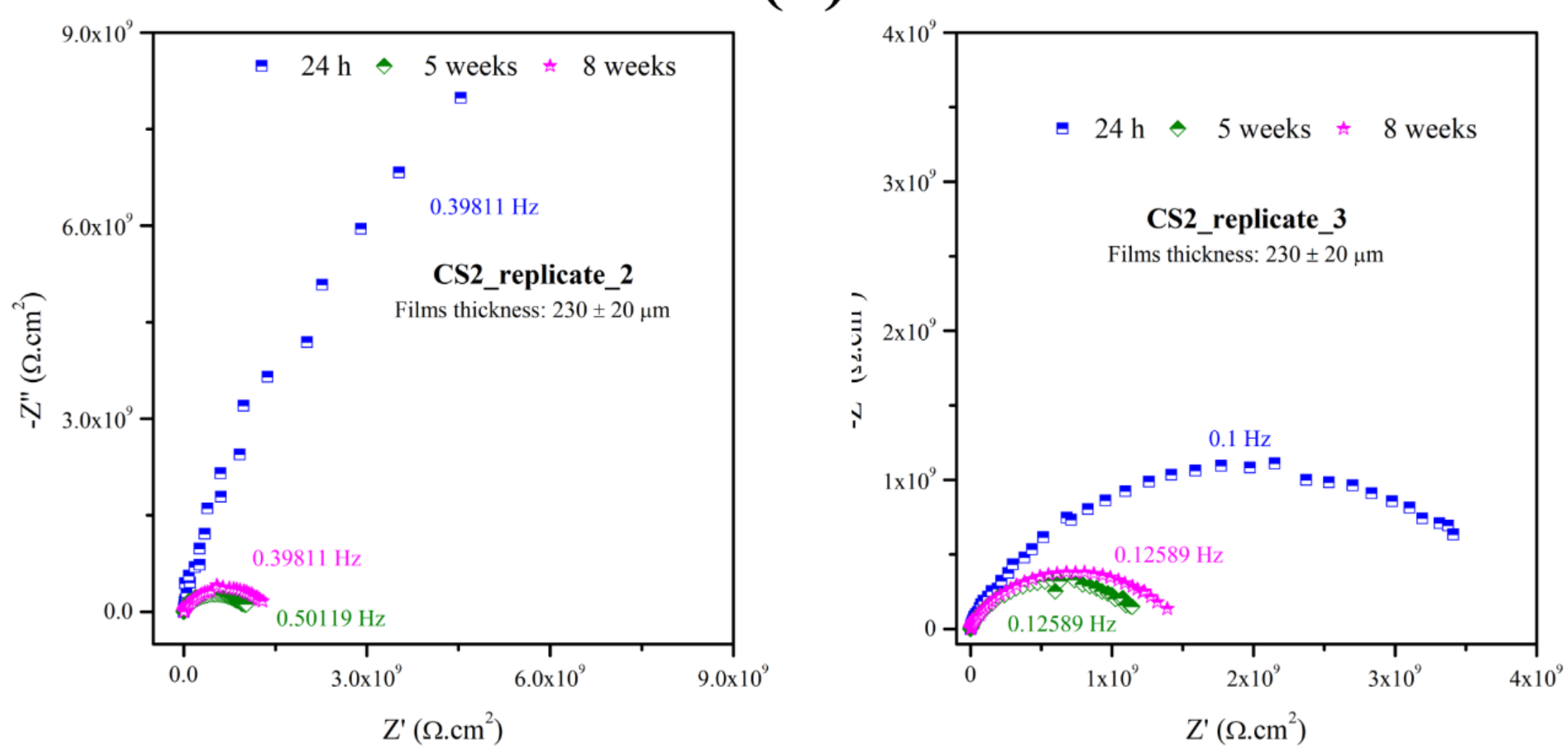

(c)

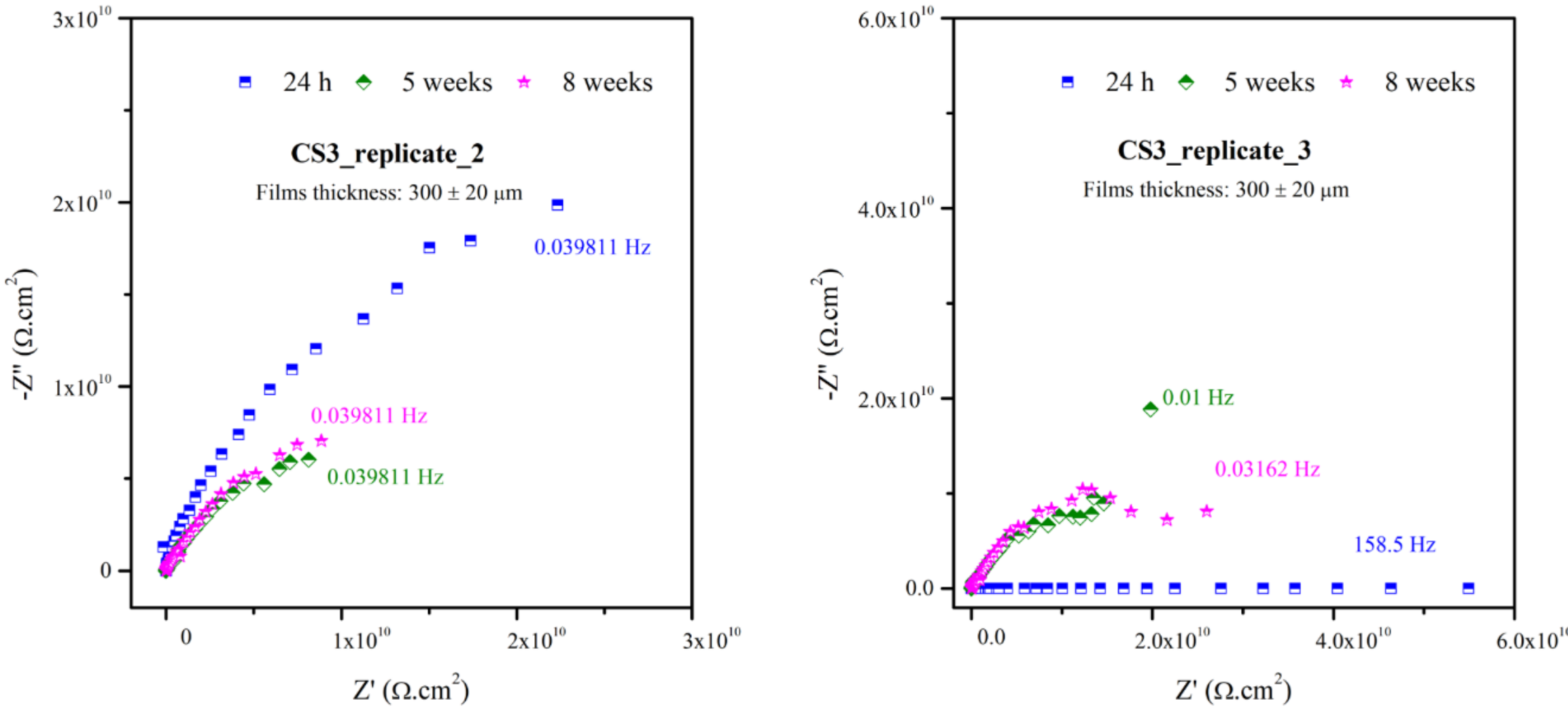

Figure S2. Nyquist plots for the other replicate of the AC-concrete (a) and AG-concrete (b) systems as a function of their immersion time in the 3.5% w/v NaCl solution. The frequency related to the maximum imaginary impedance is specified in each plot.

(a)

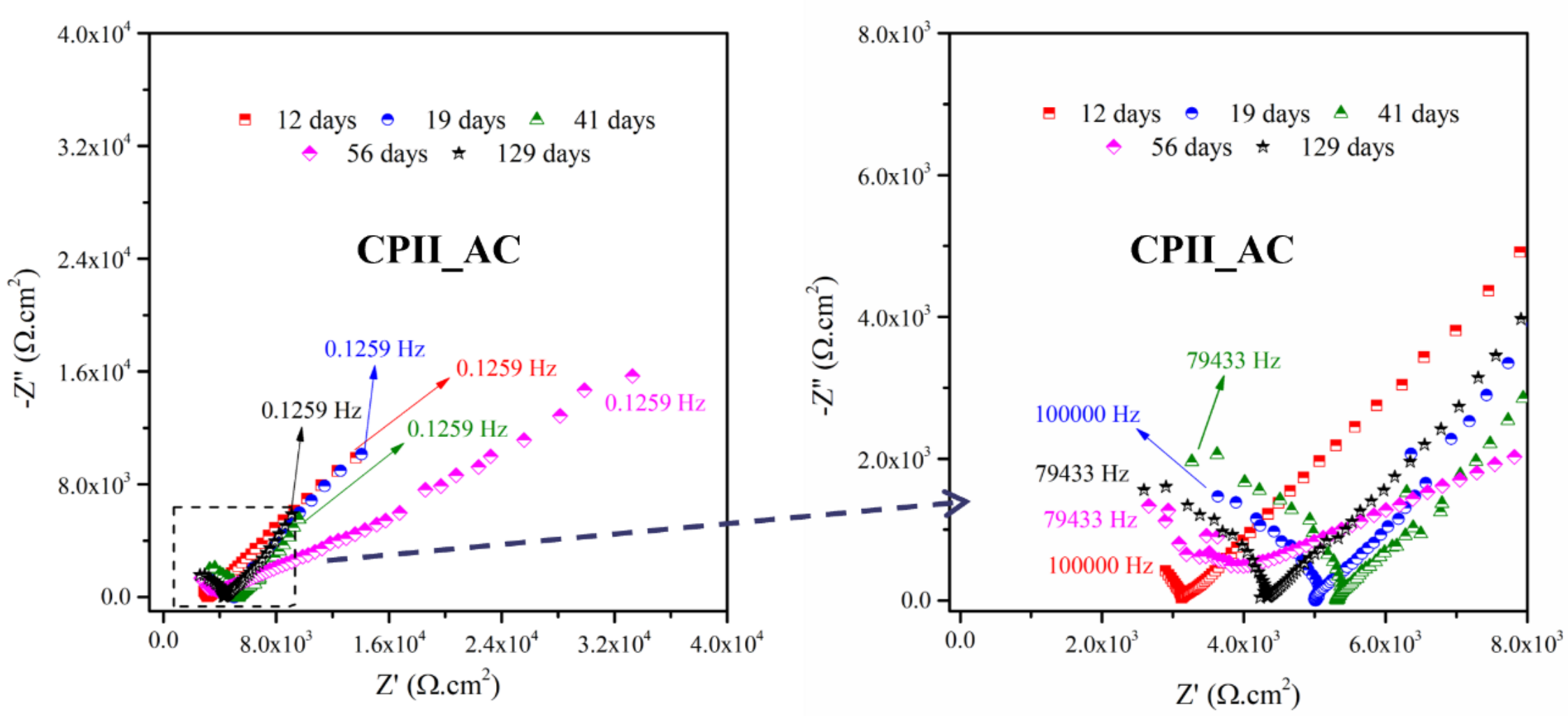

(b)

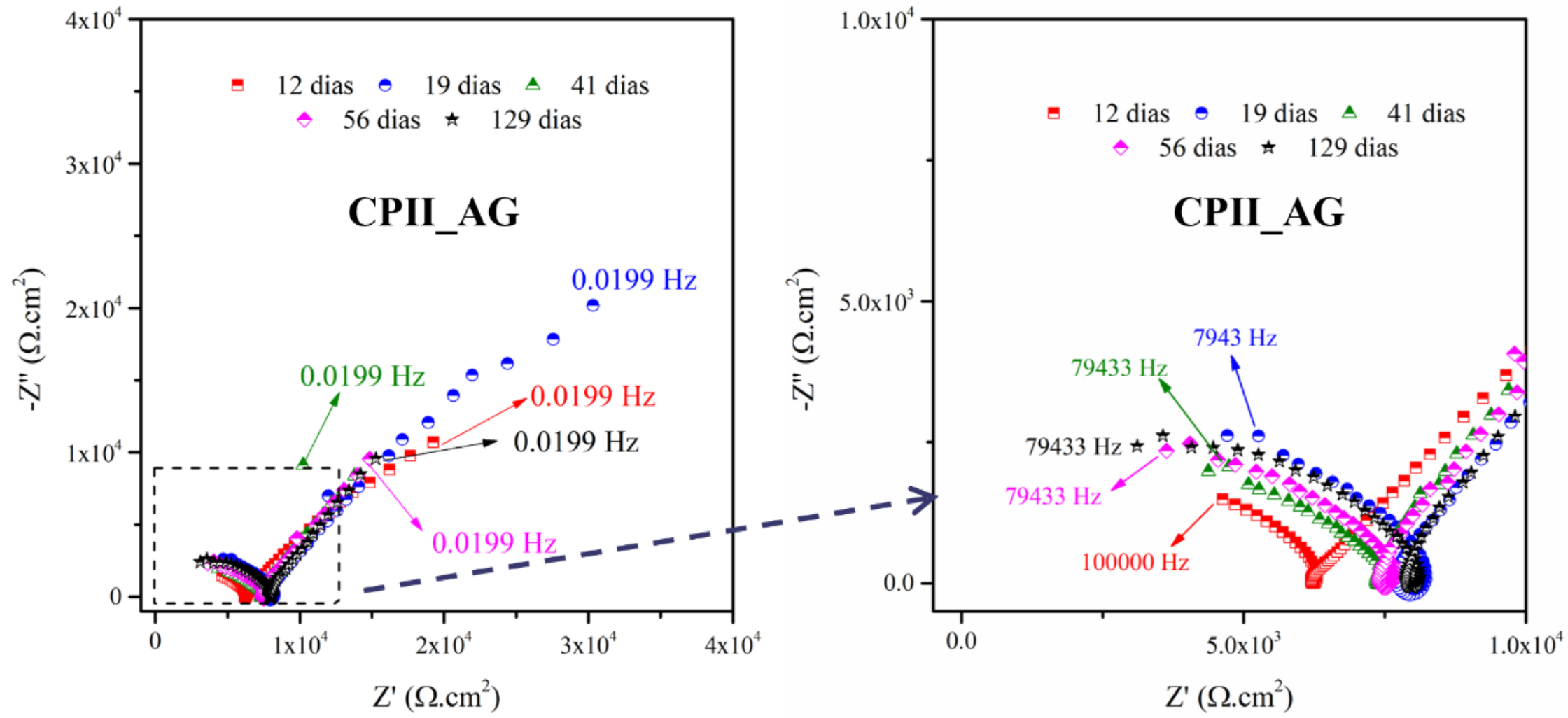

12 dias
19 dias
41 dias
56 dias
129 dias
CPII_AG
0.0199 Hz
0.0199 Hz
0.0199 Hz
0.0199 Hz
0.0199 Hz
-Z'' (Ω.cm²)
Z' (Ω.cm²)
12 dias
19 dias
41 dias
56 dias
129 dias
CPII_AG
7943 Hz
79433 Hz
79433 Hz
79433 Hz
100000 Hz
-Z'' (Ω.cm²)
Z' (Ω.cm²)
